# The impact of glycosylation on the conformational ensembles of β-, δ-, and γ-sarcoglycans

**DOI:** 10.1016/j.bpj.2026.04.018

**Published:** 2026-04-21

**Authors:** Elham Fazelpour, Gabriel A. Cook, Martin McCullagh

**Affiliations:** 1 Department of Chemistry, Oklahoma State University, Stillwater, OK 74078, USA

## Abstract

Glycosylation is a pivotal post-translational modification that influences protein folding, stability, and interactions with direct implications for muscular dystrophy pathogenesis and emerging gene therapies. Sarcoglycans (SGs), β, δ, γ, and α subunits of the dystrophin-glycoprotein complex (DGC), contain essential N-linked glycosylation sites, and mutations disrupt glycan attachment, destabilize the complex, and cause limb-girdle muscular dystrophy. However, the structural consequences of SG glycosylation remain poorly defined due to the absence of experimental SG complex structures. Here, we use homology modeling, AlphaFold predictions, and all-atom molecular-dynamics simulations to probe how N-linked glycans reshape the conformational ensembles of β-, δ-, and γ-SG monomers and the β-δ-γ heterotrimer core. We find that glycosylation increases flexibility and conformational heterogeneity in isolated monomers but reinforces a compact, stabilized architecture in the heterotrimer. Contact-map and clustering analyses show that glycans redistribute local residue interactions while preserving global trimer organization, suggesting a context-dependent role in destabilizing monomers yet reinforcing complex stability. These findings provide the first atomistic insight into how glycosylation primes SGs for assembly and may explain why mutations at glycosylation sites disrupt complex integrity and drive muscular dystrophy phenotypes.

## Significance

Sarcoglycans are membrane glycoproteins that form a core component of the dystrophin-glycoprotein complex, where they stabilize muscle cell membranes and transmit mechanical signals during contraction. Mutations that disrupt sarcoglycan glycosylation cause limb-girdle muscular dystrophies, yet the structural consequences of glycan attachment remain poorly understood because no experimental structure of the human sarcoglycan complex exists. Using homology modeling, AlphaFold predictions, and extensive all-atom molecular-dynamics simulations, we show that glycosylation reshapes sarcoglycan conformational ensembles in an assembly-dependent manner. Glycans increase conformational heterogeneity in isolated subunits but stabilize dominant conformational states within the heterotrimeric core complex. These results provide atomistic insight into how glycosylation regulates sarcoglycan stability and assembly, offering a mechanistic framework for understanding how glycosylation defects may contribute to muscular dystrophy.

## Introduction

Sarcoglycans (SGs) are single-pass transmembrane (TM) glycoproteins that form an essential sub-complex of the dystrophin-glycoprotein complex (DGC), where they maintain muscle cell integrity and transmit mechanical signals during contraction.^1^^,^^2^ Mutations in SG genes destabilize this sub-complex and cause autosomal recessive limb-girdle muscular dystrophies (LGMDs), with the loss of any one subunit often leading to degradation of the entire complex.^3^ N-linked glycosylation is introduced during folding in the endoplasmic reticulum and further processed in the Golgi and is critical for SG stability, trafficking, and membrane localization.^4^ Clinical observations underscore this importance: for example, mutations in α-SG (e.g., R77C) allow partial retention of the β-δ-γ core at the membrane, whereas loss of β- or δ-SG abolishes complex formation.^5^^,^^6^ These findings highlight the central role of the β-δ-γ core in complex stability and provide a rationale for focusing on this trimer in structural and mechanistic studies.

**Figure 1:**
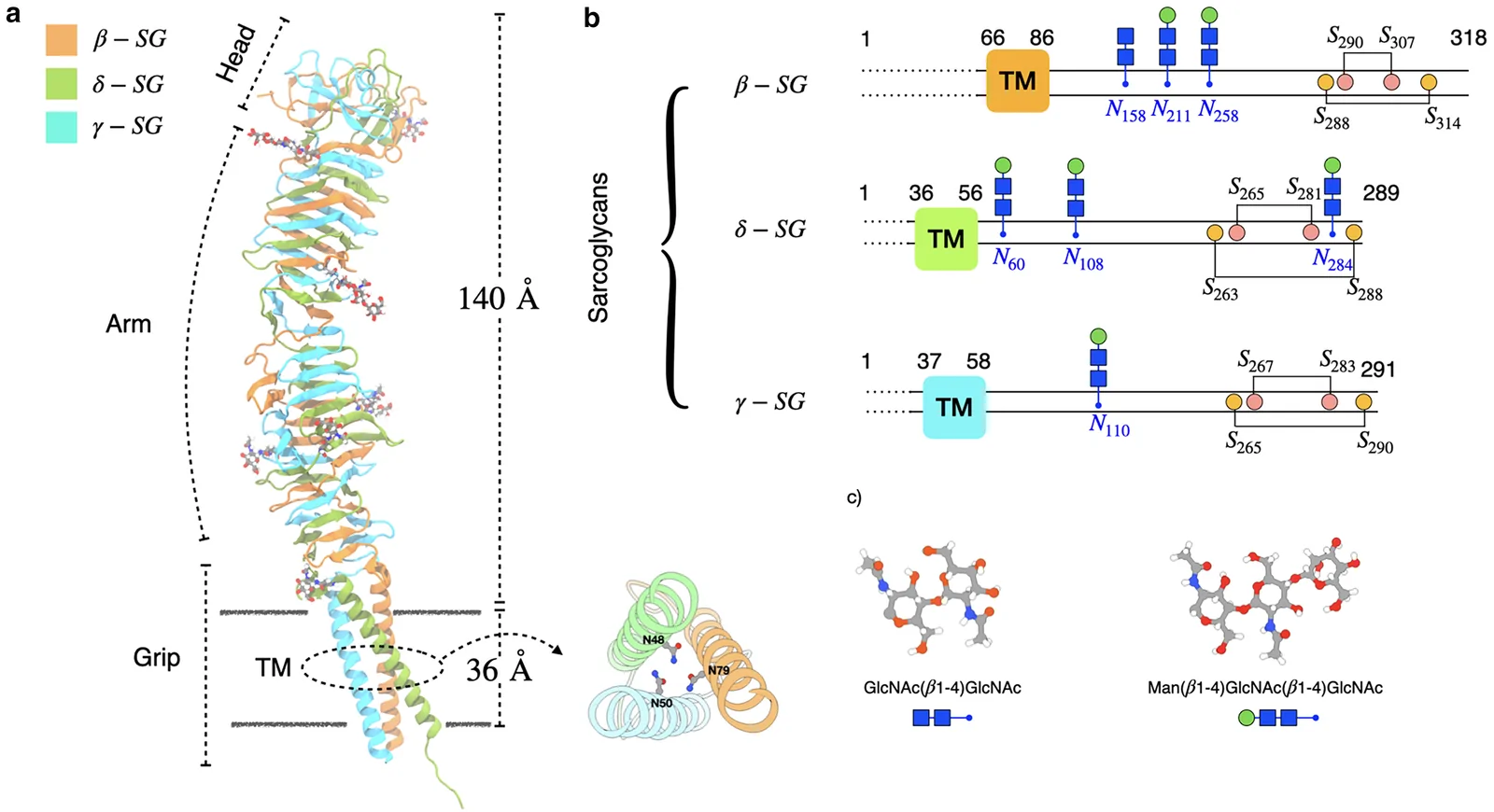
Triple β helix structure of heterotrimer SGs. (a) A boomerang-like β helix formed by β-SG-δ-SG-γ-SG. Left, the section names of the boomerang-like β helix are labeled. “Head” and “Arm” section construct the extracellular region while “Grip” contains the transmembrane (TM) and intracellular regions. Bottom right highlights a detailed extracellular-facing view of the TM domain. (b) Domain arrangement of heterotrimer SG complex. Residue numbers at domain boundaries are indicated. Modeled *N*-glycan sites and the sugar structures are shown in blue. Disulfide-bond pairs are shown in yellow and pink. Unresolved or missing residues are indicated by dashed lines for SGs. (c) The sugar structures along with their glycan codes used at *N*-glycan sites.

The structural organization of the β-δ-γ trimer reveals how these subunits form a stable platform for SG assembly. Together, these type II TM proteins adopt a boomerang-shaped architecture with three distinct regions: the “grip,” “arm,” and “head” (Figure 1A). The N-terminal TM domains form the grip, a twisted three-helix bundle stabilized primarily by hydrophobic interactions. Each subunit contains a conserved asparagine residue that mediates specific interhelical contacts (N79, N48, and N50 in β-, δ-, and γ-SG, respectively; bottom inset of Figure 1A). Beyond the membrane, extracellular β strands from all three subunits co-fold into a rigid β helix arranged in a staggered, spiral-like fashion, producing a tightly interlocked triangular architecture. The C-terminal loops of each monomer, containing conserved disulfide bonds, further stabilize both the individual subunits and the trimer by forming backbone contacts across subunits. Altogether, these cooperative interactions maintain the cohesion of the β-δ-γ core, providing a scaffold for subsequent recruitment of α-SG.^7^

Despite its central role, the contribution of glycosylation to SG folding, stability, and assembly remains poorly defined. This knowledge gap is particularly concerning in light of recent safety issues in muscular dystrophy gene therapies, such as the US Food and Drug Administration (FDA)’s suspension of Sarepta Therapeutics’ Elevidys trials following patient deaths from acute liver failure.^8^ Moreover, no experimental structure of the human SG complex currently exists, and available models are limited to isolated domains or computational predictions.^9^^,^^10^ How glycosylation modulates the conformational landscape and inter-subunit interactions of β-, δ-, and γ-SG remains largely unknown, representing a critical barrier to mechanistic understanding and therapeutic design.

Glycosylation is well established to influence protein function by altering the conformational ensemble.^11^^,^^12^^,^^13^^,^^14^ In SGs, mutation-induced changes such as the R71T substitution in δ-SG introduce new N-glycan sites and shift molecular weight, consistent with structural perturbations, while enzymatic removal of glycans from γ- and δ-SG alters apparent size and stability.^15^ These findings suggest that glycans can remodel local folding preferences and inter-subunit contacts, thereby influencing complex assembly and function.

The structural and functional consequences of N-linked glycosylation in SGs remain incompletely understood. Studies on other glycoproteins suggest that glycans can alter folding stability,^16^ dynamics,^17^ and inter-subunit interactions^18^; however, the lack of high-resolution structural data for the human SG complex has limited mechanistic insight into these effects. To address this gap, we combine homology modeling and AlphaFold-based structural predictions with extensive all-atom molecular-dynamics (MD) simulations of β-, δ-, and γ-SG monomers and their heterotrimeric core complex. We hypothesize that glycosylation modulates SG conformational ensembles by redistributing metastable states and buffering local perturbations upon complex formation. In isolated subunits, glycans increase conformational heterogeneity and sensitivity to perturbation. In contrast, within the assembled β-δ-γ trimer, glycosylation stabilizes dominant conformational states and buffers structural fluctuations, thereby pre-organizing the core complex for robust assembly and function.

### Materials and methods

#### Starting structures

Currently, no experimental structural data are available for the human SG complex. To model this structure, homologs from *Mus musculus* (PDB: 8YT8) and *Oryctolagus cuniculus* (PDB: 9C3C), which share high sequence identities with the human counterpart (91.66% and 94.54%, respectively),^9^^,^^10^ were used as templates. Guided by these homologs, the amino acid sequences of β-, δ-, and γ-SGs were extracted from UniProt^19^ (see Table S1) and modeled as both monomers and a heterotrimer using AlphaFold^20^^,^^21^ and homology modeling via the SWISS-MODEL server.^22^^,^^23^ To mimic the *in vitro* environment, all structures, whether as a monomeric or heterotrimeric system, were integrated into a 1,2-dimyristoyl-sn-glycero-3-phosphocholine (DMPC) bilayer membrane.^24^ Simulation details that differed between monomers and the heterotrimer are discussed separately.

#### SG monomeric subunit simulation preparation

Glycosylated and nonglycosylated systems of β-, δ-, and γ-SG were modeled using CHARMM36m parameters.^25^^,^^26^ Simulations were prepared with the CHARMM-GUI server,^27^^,^^28^ and each subunit was oriented according to the Orientations of Proteins in Membranes (OPM) database,^29^ with standard N- and C-terminal patches applied. Each SG subunit was embedded in a separate DMPC bilayer, solvated with TIP3P water molecules to provide a minimum 15-Å buffer above and below the membrane, and neutralized with Na^+^ and Cl^−^ ions to achieve a 0.1 M salt concentration. For glycosylated systems, CHARMM-GUI protocols were used to apply glycans at reported sites during the PDB modification step. Among the three SG subunits, δ-SG is unique in having two possible glycosylation sets: one experimentally reported site (N108, 1Glc) and three predicted sites (N60, N108, and N284, 3Glc). Details of the starting structures are provided in Tables S1–S3.

#### SG heterotrimer simulation preparation

The SG heterotrimer complex, consisting of β-, δ-, and γ-SG, was also modeled using the same protocol. For the heterotrimer, two glycosylated systems were generated corresponding to the two possible glycosylation sets of δ-SG. In the first, δ-SG was glycosylated only at N108, giving a total of five glycosylation sites in the complex (5Glc). In the second, δ-SG was glycosylated at all three predicted sites, giving a total of seven glycosylation sites (7Glc).

#### *Glycan selection and parameterization*

All experimentally confirmed N-linked glycosylation sites (Table 1) were modeled across the three SGs: β-SG at N158, N211, and N258; γ-SG at N110; and δ-SG at its predicted extracellular N-glycosylation consensus motifs (N60, N108, and N284). These sites are located on the extracellular tower formed by the β-γ-δ trimer, the surface that drives DGC assembly by providing binding sites for α-SG and dystroglycan,^10^ placing them at a structurally and functionally critical interface. For six of the seven sites, we modeled the trimannosyl core glycan (Man3; Man(β1−4)GlcNAc(β1−4)GlcNAc-ASN), the universal N-glycan core shared by all eukaryotic N-glycoforms, making it the most biologically representative and chemically conservative choice for capturing glycan-dependent effects without introducing variability from extended antennae. For β-SG N158, a diGlcNAc stub (GlcNAc(β1−4)GlcNAc-ASN) was used instead, consistent with the glycan density resolved at this site in the cryoelectron microscopy (cryo-EM) structure of the native DGC.^10^ Glycan structures were built and attached using CHARMM-GUI Glycan Reader and Modeler^27^ with the CHARMM36 carbohydrate force field.^26^ Since only N108 is experimentally confirmed in δ-SG, while N60 and N284 are computationally predicted, we simulated two δ-SG glycosylation scenarios: a minimal system retaining only the confirmed site (five glycosylated sites total, 5Glc) and a fully glycosylated system including all three predicted sites (seven sites total, 7Glc). Both scenarios were applied to the monomeric and heterotrimeric systems, enabling a direct comparison of glycan effects before and after complex formation.

**Table 1:** Glycan sites and types used in glycosylated simulations

Subunit	Glycosylation site	Glycan type
β-SG	N158	GlcNAc(β1 − 4)GlcNAc
	N211	Man(β1 − 4)GlcNAc(β1 − 4)GlcNAc
	N258	Man(β1 − 4)GlcNAc(β1 − 4)GlcNAc
δ-SG	N60	Man(β1 − 4)GlcNAc(β1 − 4)GlcNAc
	N108	Man(β1 − 4)GlcNAc(β1 − 4)GlcNAc
	N284	Man(β1 − 4)GlcNAc(β1 − 4)GlcNAc
γ-SG	N110	Man(β1 − 4)GlcNAc(β1 − 4)GlcNAc

#### MD simulation protocol

All-atom, explicit-solvent MD simulations for glycosylated and nonglycosylated SG monomeric subunits (β-SG, δ-SG and γ-SG) and SG heterotrimer complex were executed using the AMBER18 package.^25^ The cutoff distance for nonbonded interactions was set to 12 Å, after which Coulombic interactions were treated with the particle mesh Ewald method.^30^ The effects of long-range van der Waals interactions were estimated using a dispersion correction model. Periodic boundary conditions were employed. Using the equilibration and production inputs generated by CHARMM-GUI, we first ran the established seven-step minimization/equilibration process.^31^^,^^32^ Then, 20 independent 50-ns production runs were performed using the GPU-accelerated CUDA^33^ version of AMBER18, pmemd.^34^

The initial systems were minimized for 5,000 steps. Positional restraints were applied to the protein, sugars, ligands, and lipid head groups with a force constant of 10.0 kcal mol^−1^Å^−2^, while dihedral restraints were applied to sugars and lipids. Positional restraints were defined for specific residues and groups, ensuring efficient system minimization while maintaining the structural integrity of key protein and membrane regions. Then, equilibration was done using a multi-step process to gradually reduce positional restraints and stabilize the system before the production run. The initial equilibration step was over a 1-ns NVT simulation, which used a high positional restraint of 250.0 kcal mol^−1^Å^−2^ to stabilize the system while allowing the solvent to equilibrate around the protein and membrane. Over the next five equilibration steps, for a total of 8 ns, the positional restraint force constants were gradually reduced in the following order: 250.0, 100.0, 50.0, 50.0, and 25.0 kcal mol^−1^Å^−2^. This gradual reduction helps to maintain the structural integrity of the protein and other molecules while allowing the solvent and ions to equilibrate around them.

For the production simulations, we used the standard input file for NPT simulations generated by CHARMM-GUI, with temperature control using the Langevin thermostat^35^ with a friction coefficient of 1.0 ps^−1^ and semi-isotropic pressure control using the Berendsen barostat^36^ with a relaxation time of 1.0 ps. The target temperature and pressure were set to 298.15 K and 1.0 bar, respectively. Production runs of both glycosylated and nonglycosylated SG simulations (SG complex and subunits) were performed in triplicate for 1 μs each, yielding a total of 3 μs for each system. A summary of the performed simulations is provided in Table 2.

**Table 2:** Summary of MD simulations performed in β-, δ-, and γ-SG as monomer and a heterotrimer complex

System	Assembly	# Glycans	Total system size^a^	Replicas	Length per replica (μs)	Total (μs)
β-SG	monomer	0	∼173k	3	1.0	3.0
β-SG	monomer	3	∼172k	3	1.0	3.0
δ-SG	monomer	0	∼190k	3	1.0	3.0
δ-SG	monomer	1	∼190k	3	1.0	3.0
δ-SG	monomer	3	∼190k	3	1.0	3.0
γ-SG	monomer	0	∼167k	3	1.0	3.0
γ-SG	monomer	1	∼180k	3	1.0	3.0
β-δ-γ-SG	trimer	0	∼428k	3	1.0	3.0
β-δ-γ-SG	trimer	5	∼412k	3	1.0	3.0
β-δ-γ-SG	trimer	7	∼414k	3	1.0	3.0

a The number of water molecules is included in the total system size.

### Analyses

Local and global attributes of the conformational ensembles were extracted from the MD simulations. Standard analyses such as principal-component analysis, root-mean-square deviation (RMSD), root-mean-square fluctuation (RMSF), dictionary of secondary structure in proteins (DSSP), and error analysis are described in supplemental information (Section S1).

#### Conformational clustering

In this study, we employed size-and-shape space Gaussian mixture model (shape-GMM)^37^^,^^38^^,^^39^ to identify structural states (clusters) of SG proteins (individual subunits and complex) based on particle positions. The model fits multivariate Gaussian distributions to the data and estimates optimal parameters for each cluster. To determine the appropriate number of clusters, we used the elbow heuristic method alongside cross-validation (CV). The elbow heuristic method identifies the point at which adding more clusters yields diminishing improvement in log likelihood, typically marked by a minimum in its second derivative. CV was used to prevent overfitting, with five training sets generated to estimate sampling error. Frames were assigned to clusters by minimizing the Mahalanobis distance following uniform alignment. All shape-GMM analyses used a uniform product model for the covariance matrices.

#### NMR chemical-shift calculations

In this work, only the γ-SG structure experimental NMR data were available to assess the quality of the predicted structure.^24^ Given this, SHIFTX2^40^ was used to compute the backbone and side-chain ^1^H and ^15^N chemical shifts for γ-SG using the last 50 ns of the trajectory from MD simulations across all three replicas (Figure S1).

## Results and discussion

N-linked glycosylation modulates protein folding and stability through a variety of mechanisms.^41^^,^^42^^,^^43^ In the case of large glycoproteins such as SGs, glycans typically influence local conformational preferences and dampen fluctuations.^16^ In this study, we aimed to investigate the impact of glycosylation on the conformational ensemble of SG complex. Since glycosylation sites are located on the extracellular domain (ECD) region and most of the interactions happen at that region, in most of the performed analyses, we only consider the residues in extracellular region. To accomplish this, we analyze simulations of glycosylated (Glc) and nonglycosylated (NonGlc) β-, δ- and γ-SG as an isolated structure (or monomer) and heterotrimer complex. We subsequently cluster conformational ensembles using shape-GMM. The changes in the number of conformational clusters observed in monomeric SG subunits may influence how these subunits participate in SG complex assembly.

### Glycosylation impact on monomers

#### Model corroboration

To examine the influence of glycosylation on the conformational ensemble of each monomer, we initially employed SHIFTX2 NMR prediction algorithm to validate the predicted γ-SG structure (Figure S1); this analysis was limited to γ-SG, as experimental data were available only for this subunit.^24^ Subsequently, shape-GMM clustering analysis followed by principal-component analysis (PCA) were performed on both glycosylated and nonglycosylated monomers to investigate the impact of glycosylation on the diversity of conformational states. Finally, RMSFs, secondary structure (DSSP), and residue contact-map analyses were conducted to identify residue-level changes induced by glycosylation.

The comparison of experimental NMR data of NonGlc γ-SG with predicted chemical shifts reveals a generally acceptable alignment across the protein’s structure. The data in Figure S1, segmented into intracellular domain (ICD), TM domain (TMD), and ECD (see Table S1), alongside a full structure overview, show that predicted shifts cluster around 115–120 ppm (N chemical shift) and 6–8 ppm (H chemical shift), with the highest density (yellow-green) matching the concentration of experimental points (purple dots). Notably, the TMD exhibits the best agreement, with a distinct predicted cluster closely overlapping the experimental data, which is not surprising given that the helical TMD region is embedded and restrained within the DMPC bilayer membrane. The ICD, with fewer experimental points, and the broader ECD, with a wider distribution, also show reasonable consistency, although some experimental points fall outside high-density predicted areas, potentially indicating structural flexibility, post-translational modifications, or limitations in the SHIFTX2 model. Overall, SHIFTX2 provides a reliable approximation of the NMR chemical shifts for γ-SG, with the full structure analysis reinforcing this trend. The observed discrepancies, particularly in less densely sampled or more variable regions, highlight areas for further investigation, potentially requiring additional experimental data or refined predictive models.

### The ensemble view

Glycosylation reshapes the conformational ensemble by altering both the number and nature of accessible structural states. To quantify these effects, amalgamated trajectories from three replicas of each system were analyzed using conformational clustering, followed by PCA on aligned coordinates to capture the dominant modes of structural variation.

**Figure 2:**
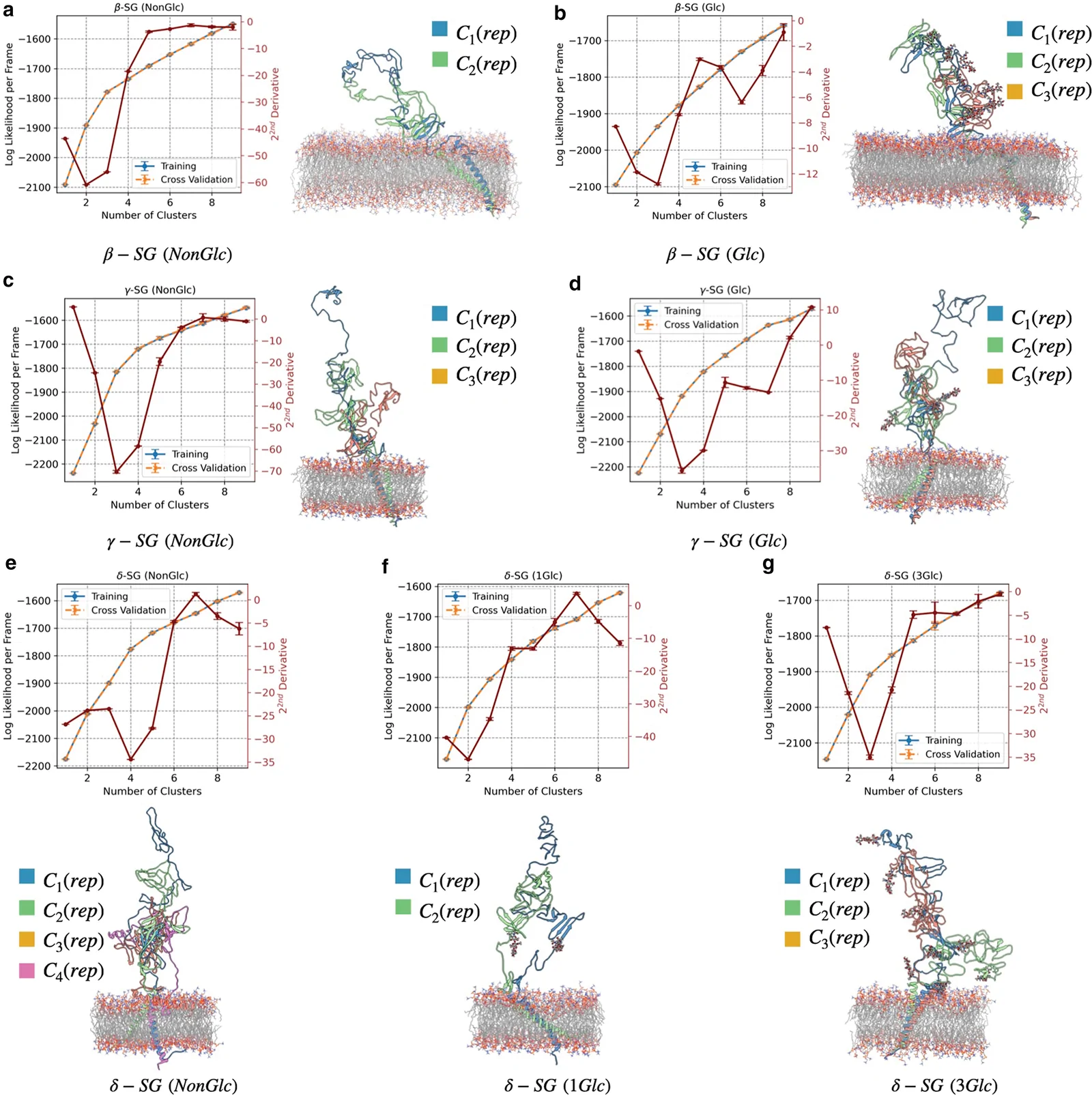
Identification of unique protein conformational clusters from amalgamated trajectories of monomeric β-SG, γ-SG, and δ-SG in Glc and NonGlc states (a–g) Each plot depicts the log likelihood per frame as a function of the number of clusters for the uniform shape-GMM. Two curves are shown in each plot: the training set (blue) and the cross-validation (CV) set (orange). Error bars represent the standard deviation obtained from sampling 10 different training sets. For each system, the representative protein structure of a cluster component is also shown and color coded accordingly, with the number of clusters ranging from a minimum of two to a maximum of four. The protein structures are superimposed to better represent the conformational differences.

**Figure 3:**
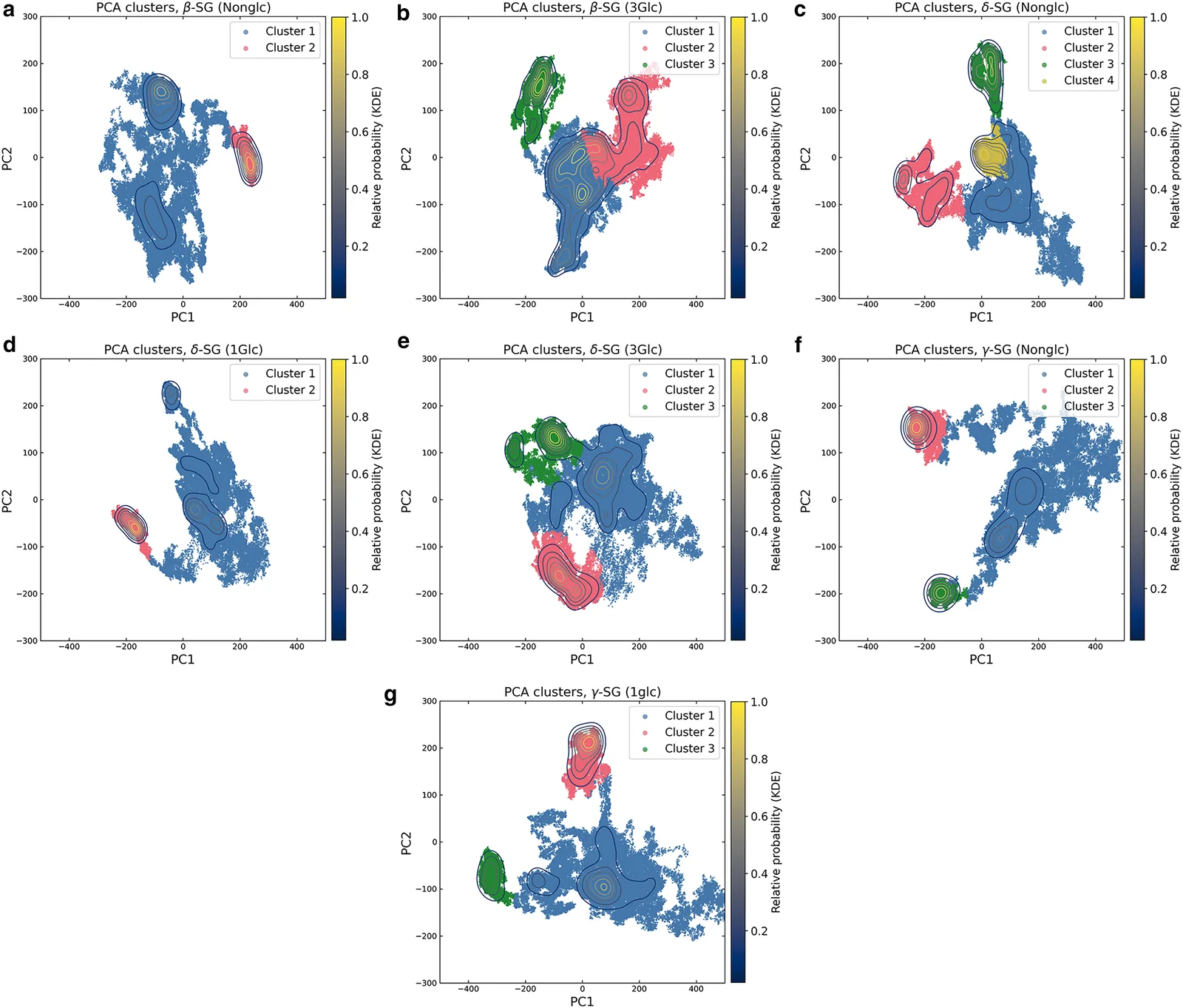
Two-dimensional PCA kernel density estimates illustrating the conformational sampling of monomers: (a) β-SG NonGlc, (b) β-SG 3Glc, (c) δ-SG NonGlc, (d) δ-SG 1Glc, (e) δ-SG 3Glc, (f) γ-SG NonGlc, and (e) γ-SG 1Glc.. Each panel represents the projection of atomic coordinates onto the first two principal components obtained from aligned MD trajectories. The color intensity reflects the probability density of sampled conformations. KDE, kernel density estimate.

For β-SG, clustering reveals pronounced differences between NonGlc and Glc systems. The NonGlc form populates two major clusters (Figure 2A; Table 3), C_1_ (76.1%) and C_2_ (23.9%), separated by a large RMSD of 11.06 Å, indicating distinct conformational states. In contrast, the Glc form samples three major clusters (Figure 2B) with substantial RMSD separation between representatives (Table 4). PCA further highlights these differences: the NonGlc system exhibits two well-separated, high-density regions broadly distributed along PC1 and PC2, reflecting high flexibility and multiple metastable states (Figure 3A). The Glc system instead displays a more compact and continuous density forming a central basin, with smoother transitions and a narrower ensemble of stable conformations (Figure 3B).

**Table 3:** Relative populations of clusters

Name	Population			
	C_1_(%)	C_2_(%)	C_3_(%)	C_4_(%)
β-SG	76.1(±14.8)	23.9(±14.8)	–	–
β-SG (Glc)	54.2(±16.9)	28.4(±11.6)	17.4(±12.9)	–
δ-SG	36.5(±14.7)	24.2(±19.4)	23.4(±16.4)	16.0(±12.9)
δ-SG (1Glc)	72.8(±16.6)	27.2(±16.6)	–	–
δ-SG (3Glc)	53.4(±21.4)	24.5(±19.0)	22.07(±18.9)	–
γ-SG	56.1(±19.5)	24.1(±19.4)	19.81(±13.2)	–
γ-SG (Glc)	57.9(±20.2)	28.6(±18.8)	13.5(±13.3)	–

± standard error of the mean, estimated via block averaging (see the supplemental information) in the monomeric SG system, calculated using shape-GMM clustering.

**Table 4:** Pairwise RMSD between cluster representative structures of monomeric SG systems with associated uncertainties (±) estimated from the standard deviation of per-frame RMSD values within each cluster

Name	RMSD (Å)					
	C_1_–C_2_	C_1_–C_3_	C_1_–C_4_	C_2_–C_3_	C_2_–C_4_	C_3_–C_4_
β-SG	11.064 (±1.630)	–	–	–	–	–
β-SG (Glc)	8.498 (±2.179)	9.309 (±1.151)	–	12.249 (±2.259)	–	–
δ-SG	11.326 (±1.869)	9.703 (±1.845)	8.555 (±1.729)	12.585 (±1.114)	11.281 (±0.908)	10.057 (±0.858)
δ-SG (1Glc)	9.370 (±2.036)	–	–	–	–	–
δ-SG (3Glc)	9.444 (±2.197)	8.925 (±2.023)	–	11.124 (±1.590)	–	–
γ-SG	14.351 (±2.874)	12.926 (±2.566)	–	13.524 (±1.721)	–	–
γ-SG (Glc)	10.929 (±2.711)	14.594 (±2.521)	–	16.365 (±1.684)	–	–

Values reflect the structural diversity within each conformational state sampled over the full 3-μs trajectory.

For δ-SG, glycosylation progressively restricts conformational diversity. The NonGlc system exhibits four distinct clusters (Figure 2E) and a broad PCA distribution with multiple high-density regions (Figure 3C), indicative of a diverse conformational ensemble. Addition of one glycosylation site (1Glc) reduces the system to two dominant clusters (Figure 2F) and produces a more concentrated density distribution (Figure 3D). Further glycosylation (3Glc) results in three clusters (Figure 2E) and an even more localized PCA density (Figure 3E). These trends indicate increasing rigidity and stabilization with increasing glycosylation.

For γ-SG, both Glc and NonGlc systems exhibit three major clusters (Figures 2C and 2D) with large inter-cluster RMSDs (Table 3), yet their conformational landscapes differ markedly. PCA reveals multimodal distributions characteristic of heterogeneous sampling; however, the Glc form displays a compact, high-density basin centered near the origin, consistent with N-linked glycan-mediated stabilization of the ECD (Figure 3G). In contrast, the NonGlc system exhibits a broader, more diffuse distribution with extended sampling along PC1, indicating increased flexibility and reduced conformational stability (Figure 3F). This behavior is consistent with experimental evidence linking incomplete glycosylation to SG destabilization and LGMD2C pathogenesis.^44^ Overall, glycosylation compacts the conformational space of γ-SG, stabilizing its native fold and supporting the structural integrity of the SG complex.

### Glycosylation impact on secondary structure

DSSP analysis was used to assess glycosylation-induced local secondary-structure changes. For β-SG, the overall distributions of α helices, β sheets, and coils are similar in Glc and NonGlc systems, indicating conserved global secondary structure. However, glycosylation induces widespread structural perturbations throughout the protein, with significant changes in secondary structure occurring at sites both proximal and distal to the glycosylation sites (GLY259, SER210, VAL155, SER260, THR209), suggesting global conformational rearrangements rather than localized effects (Figure S3). In δ-SG, both 1Glc and 3Glc systems exhibit increased persistence of helical and β sheet regions, with the 3Glc system showing the strongest stabilization, consistent with PCA results (Figures S4 and S5). Glycosylation appears to trigger allosteric correlation between modification sites and the magnitude of secondary-structure perturbation. While residues at glycosylation sites show increased coil content, regions throughout the protein domain display enhanced β sheet formation, reflecting global structural reorganization rather than local proximity effects. For γ-SG, DSSP reveals pronounced changes distributed across multiple regions of the protein. Secondary-structure perturbations occur throughout the protein fold, including but not limited to regions near the glycosylation site (N110) and within the C terminus (residues 239–265), indicating that glycosylation induces global conformational changes that propagate through allosteric mechanisms rather than direct spatial proximity effects (Figure S6).

### Differential localization and conformational effects at residue level

To assess glycosylation-induced changes in intrinsic flexibility, residue-wise RMSF profiles were computed for monomeric β-, δ-, and γ-SGs (Figure S7). As expected for the monomeric systems in solution, all isoforms exhibit elevated baseline flexibility due to the absence of stabilizing inter-subunit interactions. The three isoforms exhibit distinct glycosylation-induced responses superimposed on this elevated baseline. In β-SG, glycosylation causes a modest, global increase in flexibility without evidence of destabilization. In δ-SG, glycosylation produces region-specific effects, reducing fluctuations near the termini while increasing mobility in the central region. In contrast, γ-SG shows reduced RMSF near the glycosylation site (N110) and the C terminus, indicating glycan-mediated local stabilization that may support proper folding and complex assembly.

**Figure 4:**
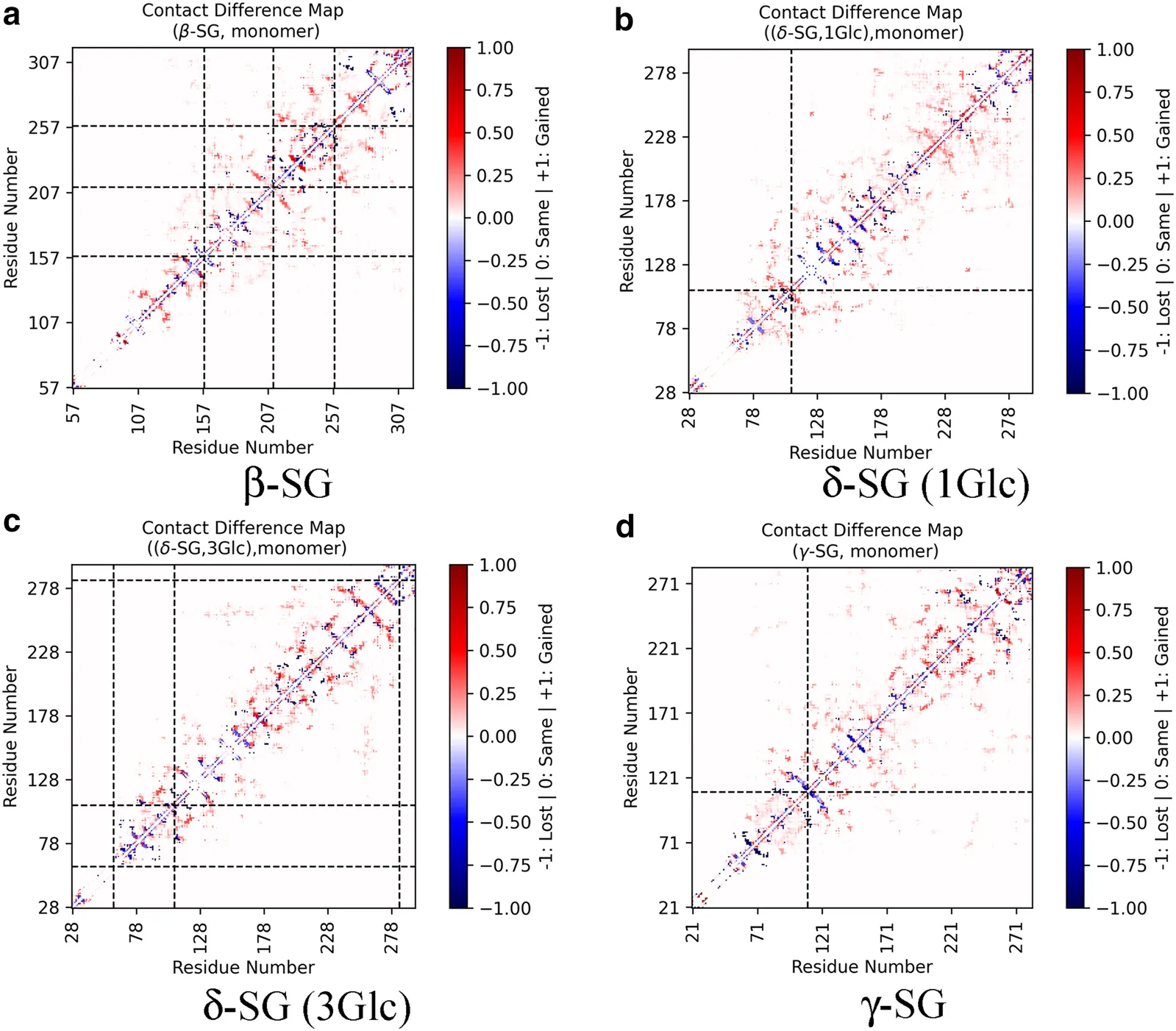
Contact-map differences between Glc and NonGlc systems for monomers: (a) β-SG, (b) δ-SG (1Glc to NonGlc), (c) δ-SG (3Glc to NonGlc), and (d) γ-SG as a monomer. Dashed lines indicate glycosylation sites. Red clusters represent new contacts or interactions formed due to glycosylation, while blue clusters indicate contacts or interactions that are lost as a result of glycosylation.

To further resolve residue-level structural effects, contact-difference maps were analyzed to identify glycosylation-induced interaction changes beyond global metrics. In monomeric β-SG, glycosylation causes widespread contact reorganization, with both gains and losses across multiple regions, indicating substantial local rearrangements (Figure 4A). Similar trends are observed in δ-SG, where glycosylation induces extensive contact changes, particularly near glycosylation sites (Figures 4B and 4C). In γ-SG, glycosylation prominently disrupts contacts in the vicinity of the glycan attachment site (Figure 4D).

In summary, glycosylation modulates SG monomers by reshaping their conformational landscapes, stabilizing dominant structural states, and reorganizing residue-level interactions in an isoform-specific manner. Ensemble analyses show that glycosylation generally compacts the conformational space most strongly in δ-SG with increasing glycosylation, while β- and γ-SG display redistribution and stabilization of distinct states, respectively. DSSP, RMSF, and contact-map analyses reveal that glycan attachment induces global changes in secondary-structure persistence, intrinsic flexibility, and residue interactions throughout the protein fold, consistent with allosteric mechanisms rather than localized proximity effects. This highlights glycosylation as a key regulator of SG structural stability and dynamics through long-range conformational reorganization.

### Glycosylation impacts on heterotrimer SG complex

Given that glycosylation shapes monomer conformations and stability, we next examine its impact on the heterotrimeric β-δ-γ complex. Glycan addition modulates inter-subunit interactions, redistributes structural fluctuations, and stabilizes dominant conformational states while preserving the overall fold of the complex.

#### Model corroboration

Given the lack of experimentally resolved structures for the human SG complex, the models were constructed using experimentally resolved *M. musculus* (PDB: 8YT8) and *O. cuniculus* (PDB: 9C3C) homologs as templates, which share high sequence identities of 91.66% and 94.54%, respectively.^9^^,^^10^ We evaluated the internal structural stability of the modeled systems using backbone RMSD as a form of model corroboration. The heterotrimeric system displays RMSD convergence after equilibration, with no evidence of progressive structural drift over the production trajectories (Figure S8). The comparable RMSD stability across components of the heterotrimeric complex indicates that the models preserve global fold integrity within the membrane environment, allowing observed differences in conformational heterogeneity and assembly-dependent behavior to be interpreted as intrinsic features of the system rather than consequences of global destabilization.

#### The ensemble view

**Figure 5:**
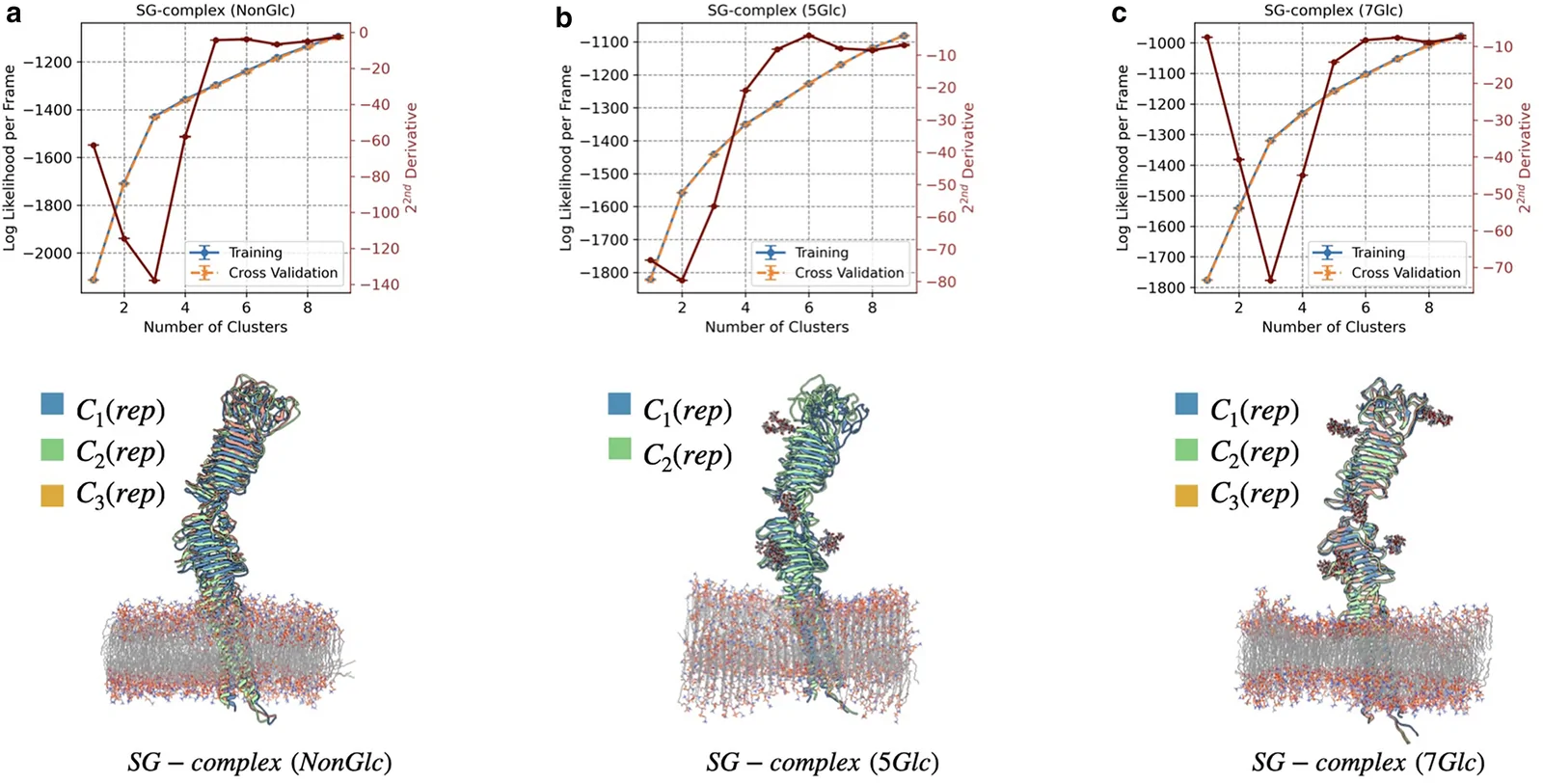
Identification of unique protein conformational clusters from amalgamated trajectories of SG complex with three subunits for three glycsolylation patterns: (a) NonGlc, (b) 5Glc, and (c) 7Glc. Each plot depicts the log likelihood per frame as a function of the number of clusters for the uniform shape-GMM. Two curves are shown in each plot: the training set (*blue*) and the cross-validation (CV) set (*orange*). Error bars represent the standard deviation obtained from sampling 10 different training sets. For each system, the representative protein structure of a cluster component is also shown and color coded accordingly, with the number of clusters ranging from a minimum of two to a maximum of three. The protein structures are superimposed to better represent the conformational differences.

**Figure 6:**
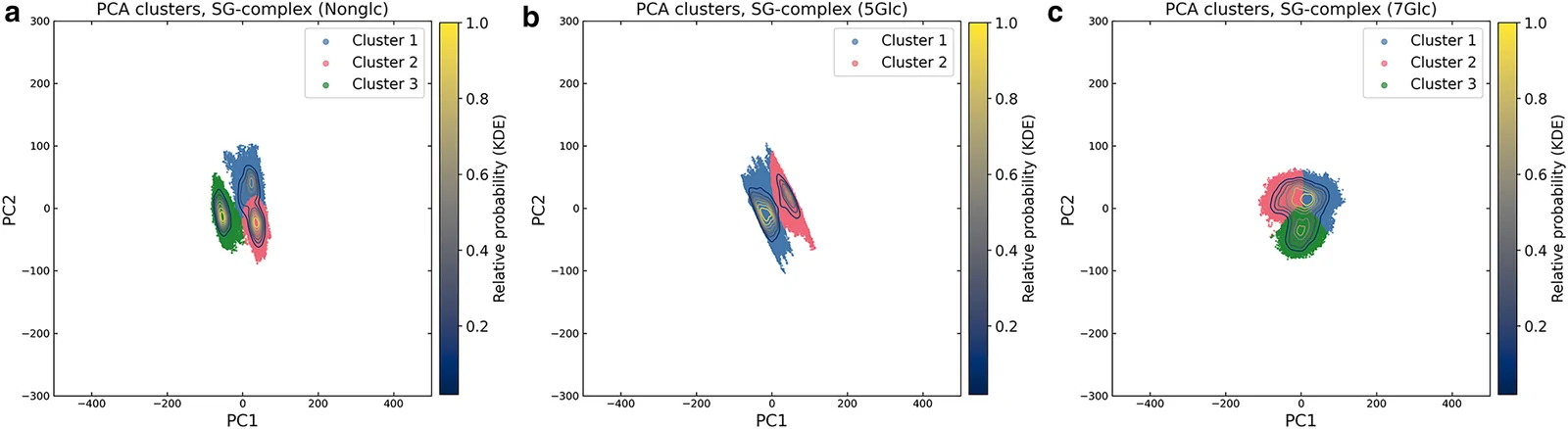
PCA-KDE maps showing conformational sampling of SG heterotrimer complex in their NonGlc and Glc states (five and seven sites) Color intensity represents conformational density along the first two principal components. Glycosylation modulates the extent and distribution of sampled conformations, indicating isoform-specific dynamic effects.

The SG complex exhibits a glycosylation-dependent reorganization of its conformational ensemble, transitioning from three clusters in the NonGlc state to two in 5Glc, and back to three in 7Glc (Figures 5A–5C; Table 5). In the NonGlc system, multiple high-density regions in the PCA map correspond to the identified clusters, with overlapping densities reflecting a flexible and heterogeneous ensemble (Figure 6A). Glycosylation at five sites leads to a single dominant high-density basin aligned with two clusters, indicating reduced conformational diversity and merging of previously distinct states (Figure 6B; Table 6). In the 7Glc system, the PCA density becomes more focused with a pronounced central peak despite the reappearance of three clusters, suggesting increased rigidity accompanied by subtle conformational sub-states arising from additional steric constraints or intramolecular interactions (Figure 6C). Overall, glycosylation initially stabilizes the complex by consolidating conformational states (NonGlc → 5Glc) followed by the emergence of nuanced structural diversity at higher glycosylation levels (7Glc). The systematic shift toward lower PC1 and PC2 values with increasing glycosylation indicates transitions between discrete cluster centroids rather than along a continuous conformational pathway.

**Table 5:** Relative populations of clusters

Name	Cluster population			
	C_1_ (%)	C_2_ (%)	C_3_ (%)	C_4_ (%)
SG complex	34.1 (±20.8)	33.0 (±18.1)	32.9 (±20.9)	–
SG complex (5Glc)	72.2 (±19.4)	27.8 (±19.4)	–	–
SG complex (7Glc)	36.6 (±13.1)	34.6 (±14.1)	28.8 (±19.0)	–

± standard error of the mean, estimated via block averaging (see the supplemental information) in heterotrimer SG complex system, calculated using shape-GMM clustering.

**Table 6:** Pairwise RMSD between cluster representative structures of heterotrimer SG systems with associated uncertainties (±) estimated from the standard deviation of per-frame RMSD values within each cluster

Name	RMSD (Å)		
	C_1_–C_2_	C_1_–C_3_	C_2_–C_3_
SG complex	1.373 (±0.223)	1.696 (±0.247)	1.899 (±0.215)
SG complex (5Glc)	1.273 (±0.315)	–	–
SG complex (7Glc)	1.234 (±0.086)	1.235 (±0.085)	1.294 (±0.083)

Values reflect the structural diversity within each conformational state sampled over the full 3-μs trajectory.

#### Glycosylation impact on secondary structure

DSSP analysis indicates that secondary-structure changes are distributed throughout the structure, with notable perturbations in the C-terminal regions (Figures S9–S14). While direct alterations near individual glycosylation sites appear minimal, the observed structural changes likely reflect the complex inter-subunit relationships within the trimer, where residues distant from one glycosylation site may be spatially proximate to glycosylation sites on neighboring subunits. This suggests that glycosylation does not disrupt the core fold or overall architecture of the SG trimer but rather exerts its influence through the three-dimensional organization of the complex, propagating conformational changes through tertiary contacts between subunits. The inter-subunit stabilization provided by the trimeric assembly constrains the large-scale conformational rearrangements observed in monomeric systems, resulting in more localized but coordinated structural adjustments. Overall, the heterotrimeric SG complex demonstrates enhanced structural stability compared to monomeric forms, with glycosylation effects manifesting through inter-subunit associations rather than isolated local perturbations.

##### Conformational effect at residue level

**Figure 7:**
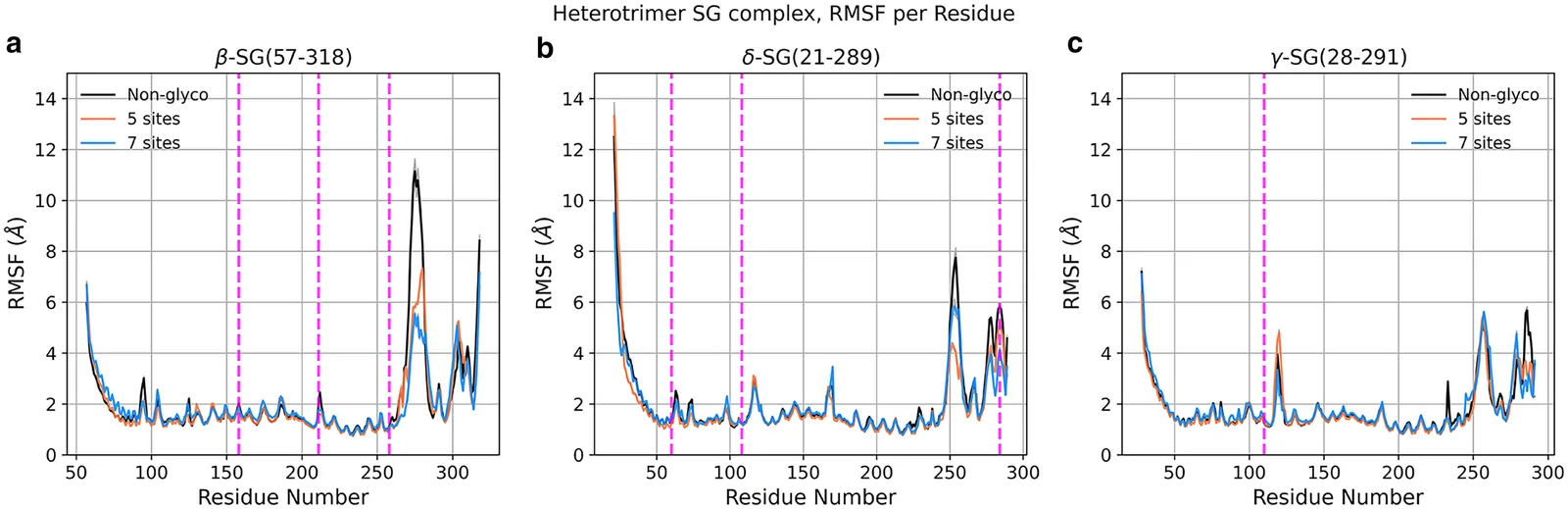
RMSF of SG heterotrimer complex. In each component, C_α_ of residues was used to calculate the RMSF. (a) belongs to β-SG, (b) to δ-SG, and (c) to γ-SG. Shaded regions represent ±standard deviation estimated from the trajectory chunking method (see Document S1.5.1).

In the heterotrimeric SG complex, interlocking of β-, δ-, and γ-SG reduces RMSF variability, with further stabilization observed upon glycosylation at five and seven sites (Figure 7), consistent with enhanced inter-subunit interactions.^5^^,^^6^ Glycosylation sites (magenta dashed lines) show no pronounced RMSF changes between NonGlc and Glc states in either monomers or the heterotrimer, indicating subtle, localized effects on flexibility. This glycan-mediated stabilization likely influences differential localization by anchoring residues in specific conformations, thereby modulating spatial organization and functional integration within the SG complex.

**Figure 8:**
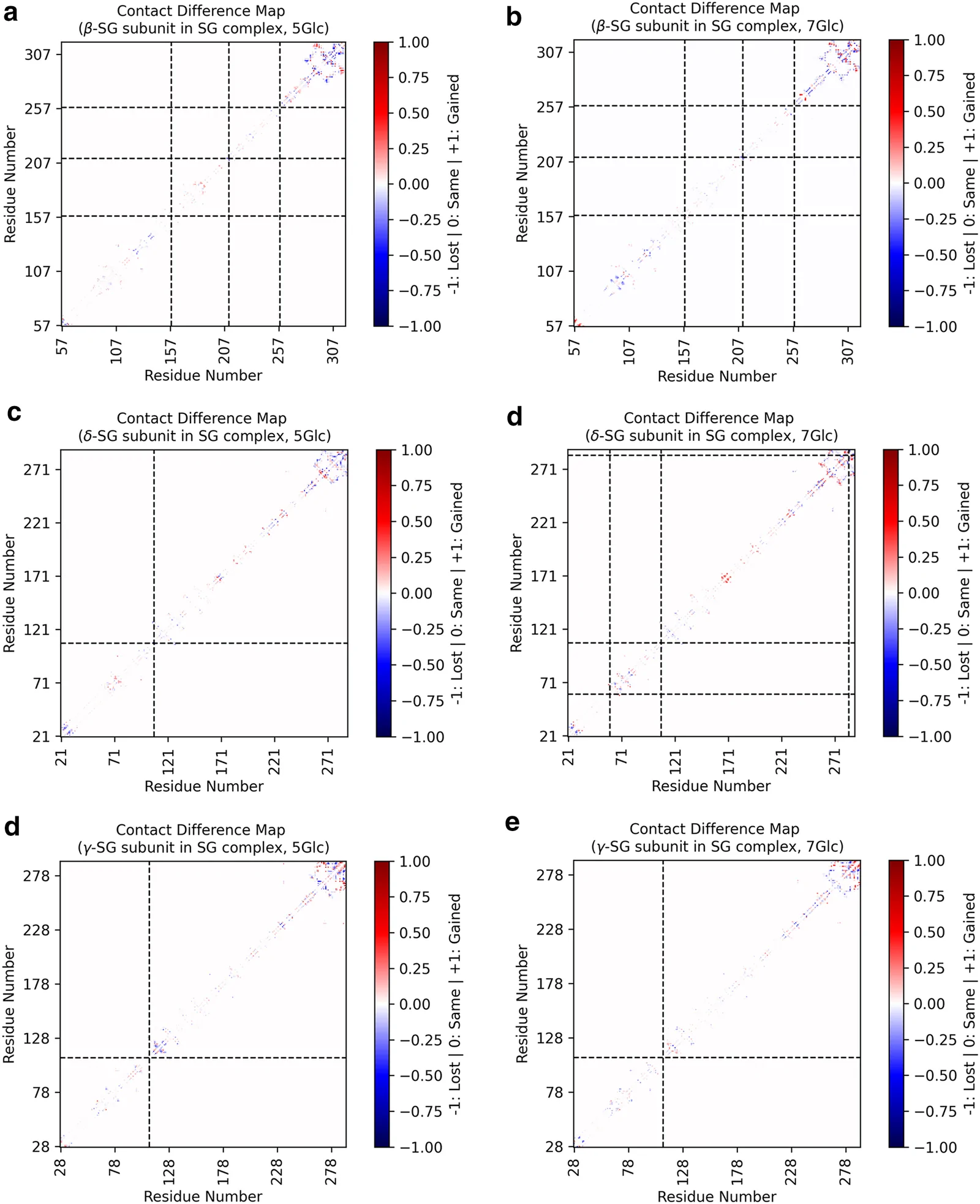
Contact-map difference plots for subunits of the SG complex between 5Glc and NonGlc (a, c, and d) and 7Glc and NonGlc (b, d, and e). Dashed lines indicate glycosylation sites. Red clusters represent new contacts or interactions formed due to glycosylation, while blue clusters indicate contacts or interactions that are lost as a result of glycosylation.

Compared to monomeric forms, incorporation of β-SG into the SG complex with 5Glc results in markedly reduced contact-map variability (Figure 8A), indicating that inter-subunit interactions buffer glycan-induced perturbations and preserve structural coherence. In contrast, the 7Glc complex shows a pronounced increase in gained off-diagonal contacts (Figure 8B), suggesting that extensive glycosylation can further reinforce structural cohesion within the complex. These trends highlight a context-dependent role of glycosylation: while glycans reorganize local contacts in isolated monomers, moderate glycosylation stabilizes core interactions in the assembled complex, and higher glycosylation promotes additional inter-residue contacts. These findings are consistent with β-SG’s proposed role in initiating SG assembly, where glycosylation may enhance local stability while perturbing distant interactions, potentially reflecting compensatory structural mechanisms relevant to LGMD2E.^45^^,^^46^ In the SG complex, contact loss is largely confined to regions near glycosylation sites and the C terminus, likely arising from secondary-structure changes or steric effects. By contrast, δ- and γ-SG exhibit only minor, C-terminal-localized contact changes, reinforcing the stabilizing role of complex assembly in accommodating glycan-induced perturbations (Figure 8C–8F).

#### Ensemble changes can have functional implications

N-glycosylation introduces bulky, flexible glycans that modulate protein structure locally and allosterically, often dampening dynamics and influencing regions distal to the modification sites.^13^ Although crystallographic characterization of glycans is challenging, complementary approaches show that glycosylation on flexible loops is particularly sensitive to environmental stress and can strongly affect allosteric communication.^47^ Beyond dynamics, N-glycosylation promotes protein folding and stability by increasing rigidity and protecting against stress, with complex glycans capable of structurally “locking” protein domains into functional orientations.^48^^,^^49^ Consistent with these principles, our results indicate that glycosylation modulates conformational heterogeneity in individual SG monomers, altering adaptability relevant to complex formation and stress response. Moreover, the SG complex itself likely mediates extracellular-to-intracellular signal transmission,^50^^,^^51^^,^^52^ with assembled subunits exhibiting distinct, more regulated flexibility compared to isolated monomers, leading to coordinated local and global structural responses.

## Conclusions

This study demonstrates that N-glycosylation modulates SG conformational behavior in a strongly assembly-dependent manner. Isolated SG monomers sample broad and heterogeneous conformational ensembles, reflecting high intrinsic flexibility that renders them sensitive to glycan-induced perturbations. Upon incorporation into the β-δ-γ heterotrimeric complex, conformational variability is markedly reduced, with glycosylation exerting more subtle effects that are buffered by inter-subunit interactions and preserve the overall architecture. Rather than inducing large structural rearrangements, glycosylation shifts the populations of metastable states, tuning local flexibility and residue-level interactions while maintaining the global fold.

These findings suggest that glycosylation acts not as a uniform stabilizer but as a context-dependent regulator that pre-organizes SG subunits for assembly, interaction, and functional resilience under stress. By explicitly linking glycan chemistry, conformational dynamics, and assembly state, this work provides a mechanistic framework for understanding how glycosylation defects may selectively destabilize SG intermediates and contribute to muscular dystrophy-associated pathologies.

## Data availability

• The data that support the findings of this study, including simulation input files, analysis scripts, and representative trajectory data, are available from the corresponding author upon reasonable request.

## Acknowledgments

The authors acknowledge the High Performance Computing Center at Oklahoma State University for providing computational resources supported in part through the 10.13039/100000001National Science Foundation grant OAC-1531128.

This work was supported by the National Institute for Allergic and Infectious Diseases of the 10.13039/100000002National Institutes of Health under award number R01AI166050.

## Author contributions

E.F. designed and performed MD simulations, carried out structural and statistical analyses, and drafted the manuscript. G.A.C. contributed to data analysis and comparison to NMR experiments. M.M. conceived and supervised the project, contributed to methodological development and data interpretation, and wrote and revised the manuscript. All authors reviewed and approved the final manuscript.

## Declaration of interests

The authors declare no competing interests.

## Supplemental information

Document S1. Figures S1–S14, Tables S1–S6, and Section S1 and S2

Document S2. Article plus supplemental information

## References

[1] Yoshida M., Ozawa E. (1990). Glycoprotein complex anchoring dystrophin to sarcolemma. J. Biochem..

[2] Ervasti J.M., Campbell K.P. (1991). Membrane organization of the dystrophin-glycoprotein complex. Cell.

[3] Holt K.H., Campbell K.P. (1998). Assembly of the sarcoglycan complex: insights for muscular dystrophy. J. Biol. Chem..

[4] Townsend D. (2014). Finding the Sweet Spot: Assembly and Glycosylation of the Dystrophin-Associated Glycoprotein Complex. Anat. Rec..

[5] Shi W., Chen Z., Chan Y.-M. (2004). Specific assembly pathway of sarcoglycans is dependent on beta-and delta-sarcoglycan. Muscle Nerve.

[6] Chan Y.-m., Bönnemann C.G., Kunkel L.M. (1998). Molecular organization of sarcoglycan complex in mouse myotubes in culture. J. Cell Biol..

[7] Chen J., Shi W., Chan Y.m.M. (2006). Identification of functional domains in sarcoglycans essential for their interaction and plasma membrane targeting. Exp. Cell Res..

[8] U.S. Food and Drug Administration (2025). FDA Requests Sarepta Therapeutics Suspend Distribution of Elevidys and Places Clinical Trials on Hold Following Three Deaths. https://t.co/lQAwI5WCOw.

[9] Liu S., Su T., Zhou Z.H. (2025). Native DGC structure rationalizes muscular dystrophy-causing mutations. Nature.

[10] Wan L., Ge X., Wu J. (2025). Structure and assembly of the dystrophin glycoprotein complex. Nature.

[11] Imperiali B., O’Connor S.E. (1999). Effect of N-linked glycosylation on glycopeptide and glycoprotein structure. Curr. Opin. Chem. Biol..

[12] Shental-Bechor D., Levy Y. (2008). Effect of glycosylation on protein folding: A close look at thermodynamic stabilization. Proc. Natl. Acad. Sci. USA.

[13] Lee H.S., Qi Y., Im W. (2015). Effects of N-glycosylation on protein conformation and dynamics: Protein Data Bank analysis and molecular dynamics simulation study. Sci. Rep..

[14] Jia Y., Liu Y., Li G. (2024). Sialylation-induced stabilization of dynamic glycoprotein conformations unveiled by time-aligned parallel unfolding and glycan release mass spectrometry. Chem. Sci..

[15] Campbell M.D., Witcher M., Michele D.E. (2016). Dilated cardiomyopathy mutations in *δ*-sarcoglycan exert a dominant-negative effect on cardiac myocyte mechanical stability. Am. J. Physiol. Heart Circ. Physiol..

[16] Wormald M.R., Dwek R.A. (1999). Glycoproteins: glycan presentation and protein-fold stability. Structure.

[17] Casalino L., Gaieb Z., Amaro R.E. (2020). Beyond Shielding: The Roles of Glycans in the SARS-CoV-2 Spike Protein. ACS Cent. Sci..

[18] Yanaka S., Sakae Y., Kato K. (2025). Exploring glycoform-dependent dynamic modulations in human immunoglobulin G via computational and experimental approaches. Proc. Natl. Acad. Sci. USA.

[19] UniProt Consortium T. (2018). UniProt: the universal protein knowledgebase. Nucleic Acids Res..

[20] Jumper J., Evans R., Hassabis D. (2021). Highly accurate protein structure prediction with AlphaFold. Nature.

[21] Varadi M., Bertoni D., Velankar S. (2024). AlphaFold Protein Structure Database in 2024: providing structure coverage for over 214 million protein sequences. Nucleic Acids Res..

[22] Waterhouse A., Bertoni M., Schwede T. (2018). SWISS-MODEL: homology modelling of protein structures and complexes. Nucleic Acids Res..

[23] Studer G., Tauriello G., Schwede T. (2021). ProMod3—A versatile homology modelling toolbox. PLoS Comput. Biol..

[24] Harris M.S., Dolan R.F., Cook G.A. (2023). In Vitro Glycosylation of the Membrane Protein *γ*-Sarcoglycan in Nanodiscs. ACS Omega.

[25] Case D.A., Berryman J.T., Kolossv P.A. (2018).

[26] Huang J., Rauscher S., MacKerell A.D. (2017). CHARMM36m: an improved force field for folded and intrinsically disordered proteins. Nat. Methods.

[27] Jo S., Kim T., Im W. (2008). CHARMM-GUI: a web-based graphical user interface for CHARMM. J. Comput. Chem..

[28] Jo S., Lim J.B., Im W. (2009). CHARMM-GUI Membrane Builder for mixed bilayers and its application to yeast membranes. Biophys. J..

[29] Lomize M.A., Lomize A.L., Mosberg H.I. (2006). OPM: orientations of proteins in membranes database. Bioinformatics.

[30] Darden T., York D., Pedersen L. (1993). Particle mesh Ewald: An *N* · log(*N*) method for Ewald sums in large systems. J. Chem. Phys..

[31] Lee J., Cheng X., Im W. (2016). CHARMM-GUI input generator for NAMD, GROMACS, AMBER, OpenMM, and CHARMM/OpenMM simulations using the CHARMM36 additive force field. Biophys. J..

[32] Jo S., Kim T., Im W. (2007). Automated builder and database of protein/membrane complexes for molecular dynamics simulations. PLoS One.

[33] Nickolls J., Buck I., Skadron K. (2008). Scalable parallel programming with cuda: Is cuda the parallel programming model that application developers have been waiting for?. ACM Queue.

[34] Case D.A., Aktulga H.M., Kollman P.A. (2021).

[35] Goga N., Rzepiela A.J., Berendsen H.J.C. (2012). Efficient algorithms for Langevin and DPD dynamics. J. Chem. Theor. Comput..

[36] Berendsen H.J.C., Postma J.P.M., Haak J.R. (1984). Molecular dynamics with coupling to an external bath. J. Chem. Phys..

[37] Klem H., Hocky G.M., McCullagh M. (2022). Size-and-shape space gaussian mixture models for structural clustering of molecular dynamics trajectories. J. Chem. Theor. Comput..

[38] Sasmal S., Pal T., McCullagh M. (2024). Quantifying Unbiased Conformational Ensembles from Biased Simulations Using ShapeGMM. J. Chem. Theor. Comput..

[39] Sasmal S., McCullagh M., Hocky G.M. (2025). Tutorial on quantifying and sampling biomolecular ensembles with ShapeGMM. J. Chem. Phys..

[40] Han B., Liu Y., Wishart D.S. (2011). SHIFTX2: significantly improved protein chemical shift prediction. J. Biomol. NMR.

[41] Shental-Bechor D., Levy Y. (2008). Effect of glycosylation on protein folding: a close look at thermodynamic stabilization. Proc. Natl. Acad. Sci. USA.

[42] Shental-Bechor D., Levy Y. (2009). Folding of glycoproteins: toward understanding the biophysics of the glycosylation code. Curr. Opin. Struct. Biol..

[43] Solá R.J., Rodríguez-Martínez J.A., Griebenow K. (2007). Modulation of protein biophysical properties by chemical glycosylation: biochemical insights and biomedical implications. Cell. Mol. Life Sci..

[44] Noguchi S., McNally E.M., Ozawa E. (1995). Mutations in the dystrophin-associated protein *γ*-sarcoglycan in chromosome 13 muscular dystrophy. Science.

[45] Tarakci H., Berger J. (2016). The sarcoglycan complex in skeletal muscle. Front. Biosci..

[46] Durbeej M., Cohn R.D., Campbell K.P. (2000). Disruption of the *β*-sarcoglycan gene reveals pathogenetic complexity of limb-girdle muscular dystrophy type 2E. Mol. Cell.

[47] Papaleo E., Saladino G., Nussinov R. (2016). The role of protein loops and linkers in conformational dynamics and allostery. Chem. Rev..

[48] Hao C., Zou Q., Shi W. (2025). Effect of glycosylation on protein folding: From biological roles to chemical protein synthesis. iScience.

[49] Rosenau J., Grothaus I.L., Waespy M. (2022). N-glycosylation modulates enzymatic activity of Trypanosoma congolense trans-sialidase. J. Biol. Chem..

[50] Barton E.R. (2006). Impact of sarcoglycan complex on mechanical signal transduction in murine skeletal muscle. Am. J. Physiol. Cell Physiol..

[51] McNally E.M., Passos-Bueno M.R., Kunkel L.M. (1996). Mild and severe muscular dystrophy caused by a single gamma-sarcoglycan mutation. Am. J. Hum. Genet..

[52] McNally E., Winder S.J. (2006). Molecular Mechanisms of Muscular Dystrophies.

