# Supplementary material for: The impact of glycosylation on the conformational ensembles of β-, δ-, and γ-sarcoglycans
Source: Biophys J. 2026 Apr 21;125(11):2657–69. doi: 10.1016/j.bpj.2026.04.018 (PMC13294987; doi:10.1016/j.bpj.2026.04.018)
Supplement: Document S1. Figures S1–S14, Tables S1–S6, and Section S1 and S2 [file mmc1.pdf]

Biophysical Journal, Volume *125*

Supplemental information

The impact of glycosylation on the conformational ensembles of β-, δ-, and γ-sarcoglycans

Elham Fazelpour, Gabriel A. Cook, and Martin McCullagh

Supporting Information For: The Impact of Glycosylation on the Conformational Ensemble of β-, δ-, and γ Sarcoglycans

Elham Fazelpour, Gabriel A. Cook, and Martin McCullagh*

Department of Chemistry, Oklahoma State University, Stillwater, OK 74078

E-mail: martin.mccullagh@okstate.edu

S1 Analyses

S1.1 Secondary Structure

The evolution of secondary structure in glycosylated and non-glycosylated systems of β-, δ-, and γ-SG (in separate simulations and as subunit of SG-complex) were analyzed using the DSSP algorithm implemented in the MDTraj Python library.[1] The analysis focused on α-helices, β-sheets, and coil content to assess glycosylation-induced changes. To quantify these changes, we compared the average percentage of each secondary structure element per residue between glycosylated and non-glycosylated systems. These per-residue differences were computed from DSSP output and visualized as bar plots. To investigate the spatial relationship between glycosylation sites and structural perturbations, residues were additionally ranked by their minimum C_α– C_α distance to any glycosylation site over the full trajectory. This distance-based ranking allowed us to examine whether secondary structure changes correlate with spatial proximity to modification sites, independent of primary sequence order. Cross-reference tables mapping distance ranks to residue numbers were generated to facilitate interpretation of the distance-ordered plots.

S1.2 Local Flexibility

Root-mean-square fluctuations (RMSFs) were calculated to assess the average fluctuation of each residue over the trajectory, providing insight into flexibility or mobility changes induced by glycosylation. RMSFs were computed using the *atomicfluct* module in cpptraj, based on the C_α atoms of each residue.[2]

S1.3 Contact difference map analysis

All contact map calculations were performed using the MDTraj Python package.[1] C_α atoms corresponding to ECD of the glycosylated and non-glycosylated systems were extracted from each trajectory. All unique C_α– C_α residue pairs were enumerated, and pairwise distances were computed for every frame with MDTraj's `compute_distances` function. The mean distance for each pair over the trajectory was calculated and binarized using a 4 Å cutoff, with distances below the cutoff considered contacts. A contact difference map was obtained by subtracting the binary contact matrix of the non-glycosylated system from that of the glycosylated system, yielding values of -1 (lost contact), 0 (unchanged), or $+1$ (gained contact). The resulting difference matrix was symmetrized, mapped to UniProt residue numbering of each SG (individually and as a subunit of SG complex), and visualized as a heatmap.

S1.4 Principal Component Analysis (PCA) of the Aligned Trajectory

The trajectories were first aligned to remove overall translational and rotational motion prior to performing Principal Component Analysis (PCA). Alignment was performed using the Shape-GMM package, which employs a maximum-likelihood uniform alignment algorithm to superimpose all frames based on the protein's geometry. Using MDAnalysis, C_α atoms of residues were selected to represent the protein backbone, and their Cartesian coordinates were extracted for all frames. The resulting coordinate array (frames $\times$ atoms $\times$ 3) was converted to a PyTorch tensor, centered by removing the center of geometry, and then aligned using `align.maximum_likelihood_uniform_alignment()` to produce a uniformly aligned trajectory. The aligned coordinates were saved as a new trajectory

file for downstream analysis. Following alignment, PCA was performed on the aligned coordinates to capture dominant modes of conformational variation. The trajectory was reshaped into a two-dimensional array (frames $\times$ atoms $\times$ 3), and PCA was applied using the scikit-learn implementation.[3] The first two principal components (PC1 and PC2) were used for visualization. Cluster assignments obtained from the Shape-GMM model were mapped onto the PCA projection to examine the relationship between identified conformational clusters and the principal components. Additionally, a kernel density estimate (KDE) was computed using the SciPy `gaussian_kde` function to visualize the density distribution of conformations in PCA space. All analyses and visualizations were performed in Python using MDAnalysis, NumPy, PyTorch, and Matplotlib.[4–7]

S1.5 Error analysis

S1.5.1 RMSF

To assess the statistical uncertainty in the root mean square fluctuation (RMSF) values, a trajectory chunking approach was employed. Each trajectory ($\approx$ 300k frames) was divided into five consecutive, non-overlapping chunks of equal length (60,000 frames each). For each chunk, an average structure was first computed and used as the reference for the RMSF calculation within that chunk. The RMSF was then computed independently for each chunk using the `atomicfluct` command in `cpptraj`.[2] The standard deviation of the per-chunk RMSF values across all five chunks was taken as the uncertainty estimate for each residue, reflecting the variability in local flexibility across different segments of the trajectory. The resulting uncertainties are shown as shaded regions (grey) in the RMSF plots.

S1.5.2 Cluster population

To assess the statistical uncertainty in cluster populations obtained from the shape-GMM clustering analysis, a trajectory chunking approach was employed. Each trajectory was divided into N consecutive, non-overlapping segments of equal length. The relative population of each cluster was computed independently within each segment as the fraction of frames assigned to that cluster. Uncertainties were estimated as the standard error of the mean of the segment-wise populations

across all N segments. This approach captures the variability in cluster populations across different portions of the trajectory and provides an estimate of statistical uncertainty. In this work, N=5 segments were used, representing a balance between sufficient sampling within each segment and a reasonable number of segments for statistical analysis.[8]

S1.5.3 Pairwise RMSD between cluster representatives

The pairwise RMSD between cluster representative structures (centroids) obtained from the shape-GMM clustering was computed using only C_α atoms. The cluster centroids were first aligned to the first centroid using uniform alignment as implemented in the `shapeGMMTorch` package.[9–11] The RMSD between each pair of aligned centroids was then calculated as:

$$\mathrm{RMSD} = \sqrt{\frac{\sum_{i=1}^{N} |\mathbf{r}_i^{(1)} - \mathbf{r}_i^{(2)}|^2}{N}} \tag{1}$$

where $\mathbf{r}_i^{(1)}$ and $\mathbf{r}_i^{(2)}$ are the coordinates of the i-th C_α atom in the two centroids being compared and N is the total number of C_α atoms. To estimate the uncertainty in the RMSD between each pair of centroids, the intra-cluster structural spread was quantified by computing the RMSD of every trajectory frame assigned to a given cluster relative to its centroid. The standard deviation of these per-frame RMSD values, σ_i, was taken as a measure of the structural diversity within cluster i. The combined uncertainty for the pairwise RMSD between clusters i and j was then propagated in quadrature as:

$$\sigma_{ij} = \sqrt{\sigma_i^2 + \sigma_j^2} \tag{2}$$

S2 Supporting Tables Figures

Table S1: Summary of model building of the sarcoglycan complex

subunit	Length(aa) /Uniprot ID	Modeled regions	Domains	Modifications	
				Glycosylation	Disulfide bonds
β-sarcoglycan	318 /Q16585	57-318	TM:66-86 ECD:87-318	N158, N211, N258	C288-C314 C290-C307
δ-sarcoglycan	289 /Q92629	21-289	TM:36-56 ECD:57-289	N60, N108, N284	C263-C288 C265-C281
γ-sarcoglycan	291 /Q13326	28-291	TM:37-58 ECD:59-291	N110	C265-C290 C267-C283

Table S2: Detailed information about the simulations of both the sarcoglycan subunits and the heterotrimer complex generated using CHARMM-GUI. For the sarcoglycan subunits, the glycosylated and non-glycosylated systems followed the same protocol.

subunit	Box Size (Å)	# of DMPC	Tilt angle	[NaCl]
β-SG	X:90 Y:90 Z:222	250	39°	0.1M
δ-SG	X:90 Y:90 Z:242	250	36°	0.1M
γ-SG	X:90 Y:90 Z:231	250	28°	0.1M
heterotrimer complex (non-glycosylated)	X:129 Y:129 Z:271	250	1°	0.1M
heterotrimer complex (glycosylated)	X:129 Y:129 Z:261	250	1°	0.1M

Table S3: Glycan types at each glycosylation site in glycosylated simulations

subunit	Glycolation site	Glycan type
	N158	GlcNAc(b1-4)GlcNAc
β-SG	N211	Man(b1-4)GlcNAc(b1-4)GlcNAc
	N258	Man(b1-4)GlcNAc(b1-4)GlcNAc
	N60	Man(b1-4)GlcNAc(b1-4)GlcNAc
δ-SG	N108	Man(b1-4)GlcNAc(b1-4)GlcNAc
	N284	Man(b1-4)GlcNAc(b1-4)GlcNAc
γ-SG	N110	Man(b1-4)GlcNAc(b1-4)GlcNAc

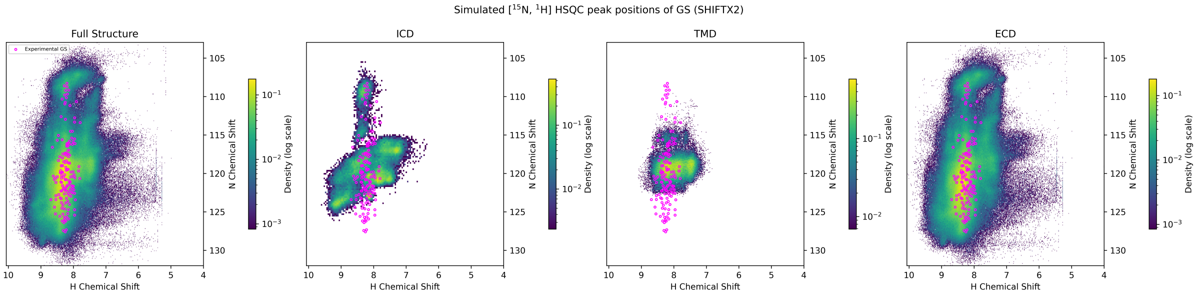

(a) Replica 1

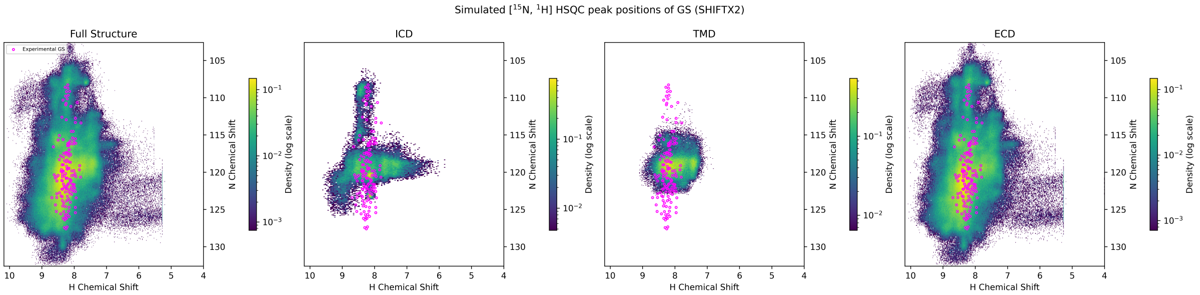

(b) Replica 2

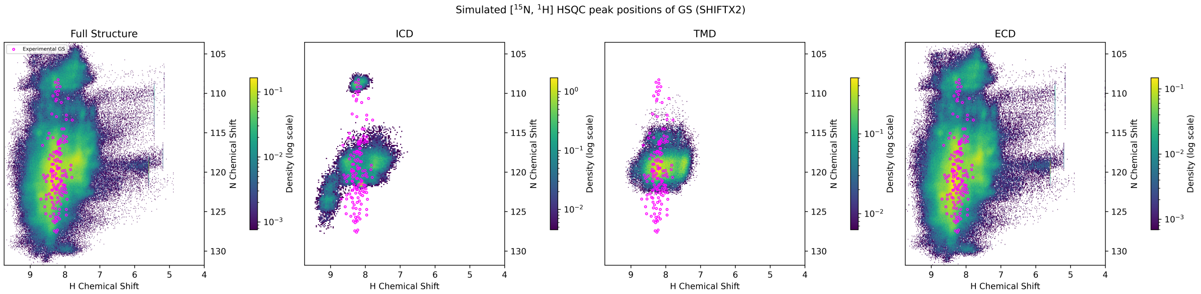

(c) Replica3

Figure S1: Backbone and side chain ^{1}H and ^{15}N chemical shifts of γ-SG using SHIFTX2 and comparison with experimental NMR data (purple dots)[12]

Table S4: RMSF differences at the glycosylation site in isolated SG subunits.
$\Delta\text{RMSF}_i = \text{RMSF}_{i,P} - \text{RMSF}_{i,GP}$, where i is for the glycosylated site, **GP** for glycosylated protein, and **P** for the nonglycosylated protein.

Name	# of glycans	Glycosylation site	# of sugar residues	Location of glycosylation site	ΔRMSF_i	Avg. $\mid\Delta\text{RMSF}\mid$
		ASN-158	2	loop	1.4812	
β-SG	3	ASN-211	3	loop	-1.3958	1.1478
		ASN-258	3	β-sheet	-0.5664	
		ASN-108		loop	-0.6662	
δ-SG	1	-	3	loop	-	-
		-		loop	-	
		ASN-60	3	loop	-0.0446	
δ-SG	3	ASN-108	3	loop	-1.4335	0.5337
		ASN-284	3	loop	0.1231	
γ-SG	1	ASN-110	3	loop	-3.6695	-

Table S5: Glycosylation site information and RMSF differences at the glycosylation site, when 5 of 7 reported glycosylation sites are glycosylated.
$\Delta\text{RMSF}_i = \text{RMSF}_{i,P} - \text{RMSF}_{i,GP}$, where i is for the glycosylated site, **GP** for glycosylated protein, and **P** for the nonglycosylated protein.

Name	# of glycans	Glycosylation site	# of sugar residues	Location of glycosylation site	ΔRMSF_i	Avg. $\mid\Delta\text{RMSF}\mid$
		ASN-158	2	loop	0.1647	
β-SG	3	ASN-211	3	loop	0.439	0.2232
		ASN-258	3	β-sheet	0.0661	
		ASN-108	3	loop	-0.0065	
δ-SG	1	-	-	-	-	-
		-	-	-	-	
γ-SG	1	ASN-110	3	loop	-0.0352	-

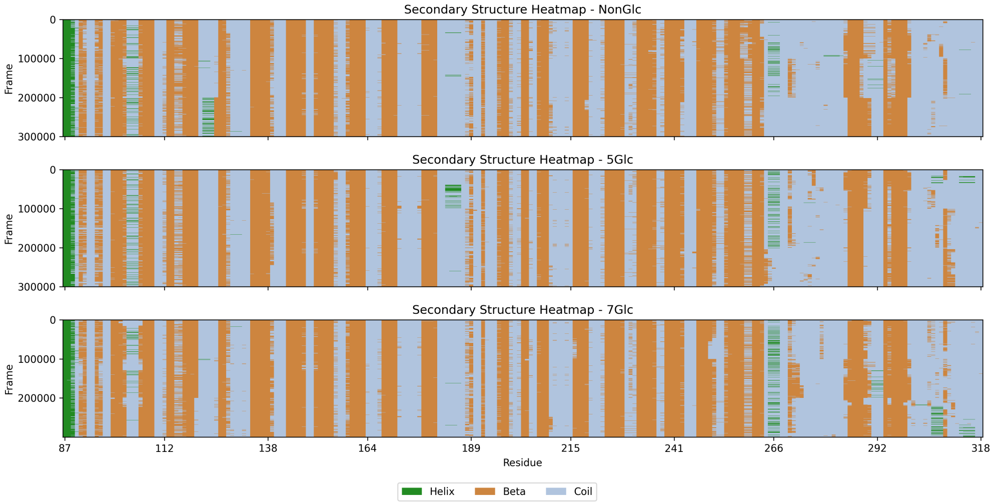

(a) ECD of β-SG in the SG complex

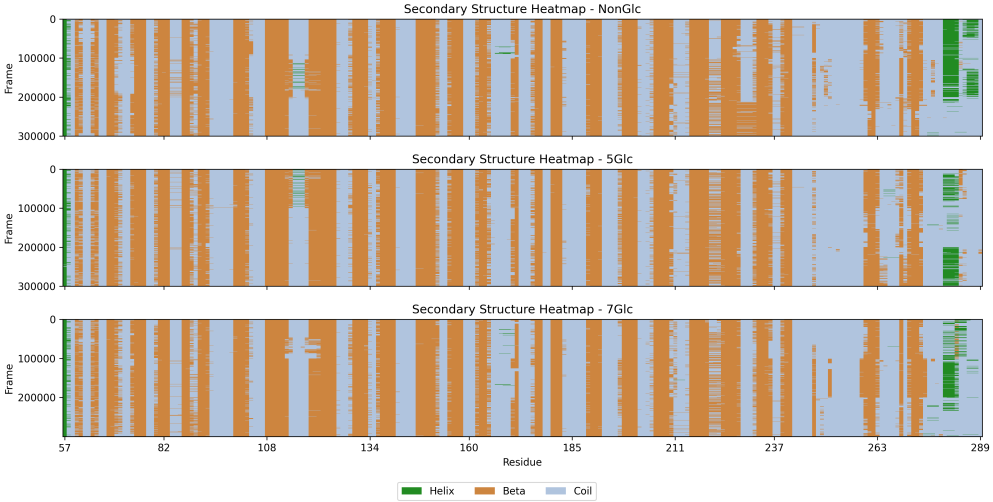

(b) ECD of δ-SG in the SG complex

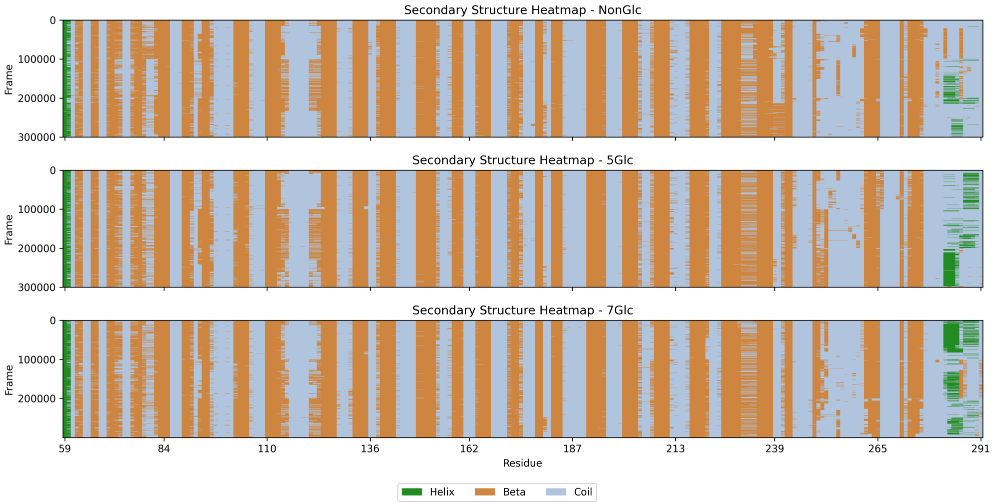

(c) ECD of γ-SG in the SG complex

Figure S2: Secondary structure (DSSP) analysis of the SG complex components under nonglycosylated and glycosylated (5Glc and 7Glc) conditions over 3μs of accumulated data.

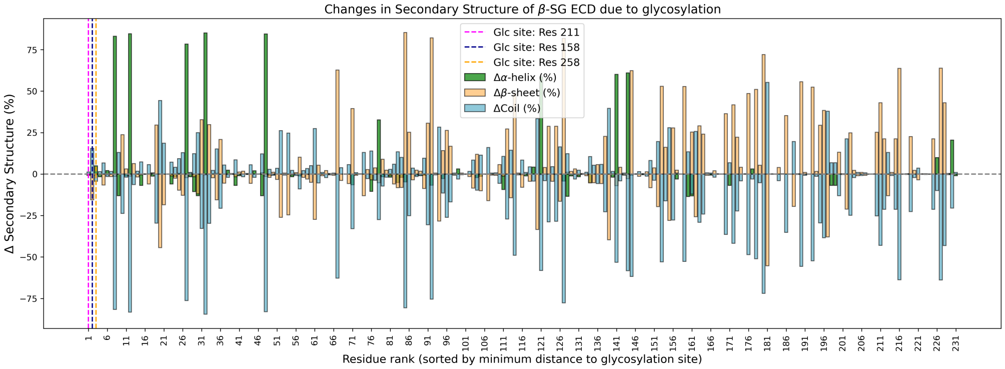

Rank — Residue Cross-Reference Table

Rank	Residue	Rank	Residue	Rank	Residue	Rank	Residue
1	211	59	214	117	305	175	172
2	158	60	133	118	295	176	226
3	258	61	255	119	308	177	307
4	139	62	237	120	205	178	219
5	212	63	236	121	276	179	299
6	210	64	160	122	177	180	231
7	257	65	309	123	284	181	223
8	259	66	289	124	114	182	148
9	157	67	217	125	215	183	186
10	159	68	181	126	203	184	170
11	194	69	318	127	197	185	187
12	262	70	296	128	168	186	220
13	151	71	200	129	290	187	94
14	129	72	240	130	230	188	202
15	244	73	302	131	229	189	115
16	239	74	182	132	152	190	147
17	136	75	272	133	238	191	312
18	291	76	252	134	265	192	300
19	128	77	283	135	207	193	146
20	234	78	264	136	297	194	314
21	293	79	227	137	192	195	145
22	282	80	281	138	127	196	126
23	246	81	249	139	204	197	222
24	137	82	286	140	279	198	103
25	134	83	266	141	275	199	105
26	287	84	253	142	176	200	110
27	263	85	198	143	183	201	313
28	138	86	179	144	274	202	221
29	251	87	288	145	196	203	99
30	155	88	268	146	149	204	95
31	233	89	269	147	267	205	117
32	261	90	248	148	165	206	101
33	150	91	112	149	131	207	116
34	235	92	199	150	206	208	93
35	135	93	304	151	311	209	100
36	303	94	191	152	190	210	109
37	243	95	277	153	232	211	119
38	208	96	216	154	111	212	106
39	140	97	241	155	163	213	125
40	245	98	184	156	178	214	107
41	193	99	306	157	201	215	102
42	273	100	166	158	130	216	118
43	132	101	164	159	218	217	98
44	113	102	254	160	167	218	120
45	209	103	301	161	169	219	96
46	310	104	278	162	189	220	92
47	156	105	292	163	298	221	124
48	260	106	175	164	144	222	108
49	270	107	162	165	188	223	121
50	280	108	161	166	185	224	97
51	213	109	316	167	153	225	91
52	256	110	285	168	173	226	88
53	141	111	250	169	174	227	123
54	271	112	154	170	224	228	122
55	228	113	242	171	104	229	87
56	180	114	143	172	225	230	89
57	195	115	142	173	315	231	90
58	294	116	247	174	171		

Figure S3: Glycosylation-induced changes in secondary structure of monomer β-SG ECD. Difference in secondary structure content (Δ%) between glycosylated (3 Glc) and non-glycosylated systems (glycosylated - non-glycosylated) per residue, assigned using the DSSP algorithm. Residues are ranked by their minimum C_α– C_α distance to the glycosylation site (N158, N211 and N258) over the 3μs trajectory. The cross-reference table provides the correspondence between rank and residue number.

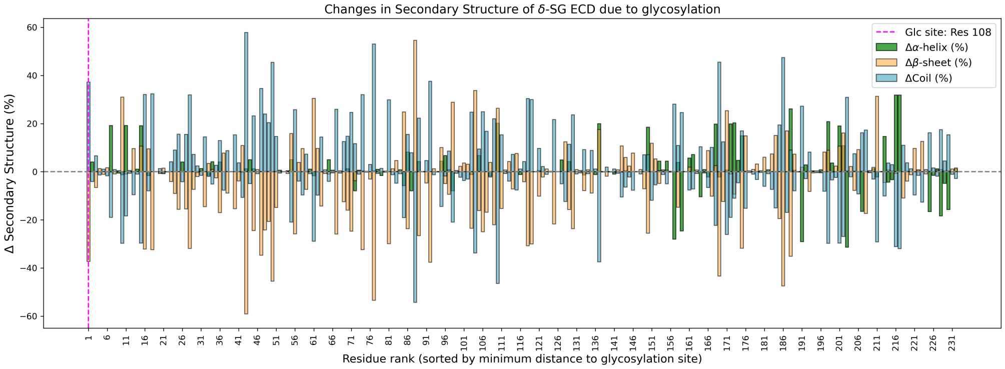

Rank — Residue Cross-Reference Table

Rank	Residue	Rank	Residue	Rank	Residue	Rank	Residue
1	108	59	67	117	199	175	240
2	127	60	142	118	173	176	215
3	107	61	82	119	198	177	115
4	109	62	85	120	122	178	289
5	80	63	96	121	174	179	250
6	126	64	135	122	181	180	214
7	99	65	93	123	185	181	221
8	106	66	159	124	200	182	190
9	90	67	68	125	147	183	261
10	101	68	158	126	184	184	249
11	100	69	133	127	194	185	259
12	79	70	157	128	180	186	217
13	105	71	69	129	223	187	251
14	143	72	153	130	61	188	239
15	98	73	167	131	120	189	231
16	125	74	164	132	193	190	242
17	102	75	202	133	229	191	286
18	130	76	204	134	186	192	266
19	72	77	165	135	60	193	269
20	89	78	161	136	63	194	213
21	88	79	83	137	207	195	258
22	110	80	123	138	179	196	263
23	132	81	172	139	114	197	260
24	70	82	226	140	121	198	209
25	71	83	65	141	230	199	265
26	128	84	160	142	222	200	244
27	91	85	84	143	248	201	210
28	170	86	197	144	175	202	212
29	78	87	152	145	188	203	287
30	104	88	141	146	271	204	273
31	144	89	224	147	177	205	216
32	95	90	203	148	119	206	241
33	103	91	182	149	270	207	282
34	81	92	149	150	238	208	243
35	87	93	201	151	272	209	267
36	129	94	112	152	176	210	257
37	131	95	225	153	246	211	220
38	169	96	195	154	192	212	116
39	73	97	166	155	247	213	211
40	134	98	151	156	187	214	276
41	154	99	66	157	285	215	275
42	97	100	137	158	264	216	234
43	156	101	140	159	284	217	235
44	94	102	146	160	59	218	268
45	155	103	64	161	283	219	232
46	86	104	136	162	57	220	254
47	163	105	196	163	178	221	252
48	76	106	183	164	58	222	274
49	75	107	138	165	191	223	233
50	171	108	228	166	219	224	117
51	74	109	139	167	118	225	281
52	168	110	206	168	236	226	278
53	111	111	148	169	218	227	255
54	162	112	113	170	262	228	279
55	92	113	227	171	189	229	277
56	77	114	205	172	237	230	280
57	124	115	150	173	208	231	253
58	145	116	62	174	245	232	256

Figure S4: Glycosylation-induced changes in secondary structure of monomer δ-SG ECD. Difference in secondary structure content ($\Delta\%$) between glycosylated (1 Glc) and non-glycosylated systems (glycosylated - non-glycosylated) per residue, assigned using the DSSP algorithm. Residues are ranked by their minimum C_α– C_α distance to the glycosylation site (N108) over the 3μs trajectory. The cross-reference table provides the correspondence between rank and residue number.

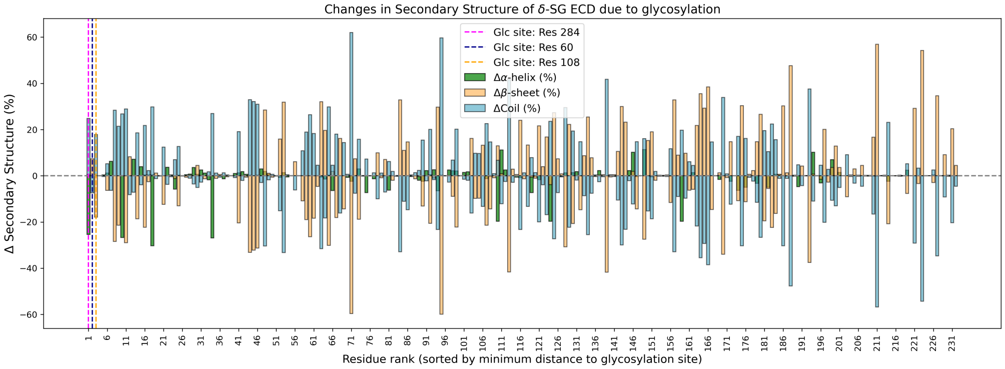

Rank — Residue Cross-Reference Table

Rank	Residue	Rank	Residue	Rank	Residue	Rank	Residue
1	284	59	71	117	254	175	147
2	60	60	172	118	268	176	196
3	108	61	70	119	277	177	231
4	127	62	129	120	93	178	247
5	59	63	89	121	102	179	237
6	107	64	103	122	124	180	246
7	283	65	77	123	150	181	137
8	109	66	83	124	153	182	195
9	61	67	163	125	132	183	114
10	285	68	155	126	157	184	176
11	75	69	165	127	267	185	245
12	170	70	266	128	173	186	160
13	57	71	218	129	271	187	191
14	74	72	65	130	174	188	221
15	281	73	211	131	79	189	188
16	67	74	250	132	91	190	194
17	169	75	276	133	135	191	228
18	287	76	72	134	269	192	226
19	126	77	63	135	141	193	259
20	252	78	149	136	115	194	206
21	95	79	104	137	212	195	256
22	280	80	145	138	249	196	236
23	66	81	275	139	272	197	258
24	84	82	130	140	159	198	232
25	96	83	263	141	190	199	235
26	73	84	140	142	136	200	233
27	97	85	154	143	139	201	227
28	99	86	162	144	138	202	122
29	58	87	94	145	142	203	225
30	100	88	274	146	207	204	120
31	168	89	278	147	92	205	234
32	106	90	219	148	241	206	257
33	282	91	143	149	208	207	193
34	286	92	216	150	255	208	113
35	171	93	210	151	248	209	177
36	253	94	82	152	240	210	223
37	62	95	217	153	123	211	222
38	125	96	158	154	189	212	178
39	251	97	167	155	119	213	224
40	215	98	166	156	239	214	197
41	105	99	78	157	146	215	121
42	86	100	111	158	262	216	185
43	214	101	213	159	152	217	184
44	68	102	80	160	133	218	187
45	69	103	131	161	229	219	205
46	76	104	118	162	175	220	180
47	279	105	164	163	192	221	199
48	87	106	148	164	270	222	198
49	98	107	64	165	261	223	200
50	110	108	264	166	161	224	179
51	85	109	117	167	260	225	186
52	101	110	151	168	242	226	204
53	88	111	209	169	230	227	182
54	265	112	144	170	243	228	203
55	116	113	273	171	244	229	183
56	128	114	134	172	220	230	202
57	289	115	90	173	112	231	201
58	156	116	81	174	238	232	181

Figure S5: Glycosylation-induced changes in secondary structure of monomer δ-SG ECD. Difference in secondary structure content (Δ%) between glycosylated (3 Glc) and non-glycosylated systems (glycosylated - non-glycosylated) per residue, assigned using the DSSP algorithm. Residues are ranked by their minimum C_α– C_α distance to the glycosylation site (N60, N108, N284) over the 3μs trajectory. The cross-reference table provides the correspondence between rank and residue number.

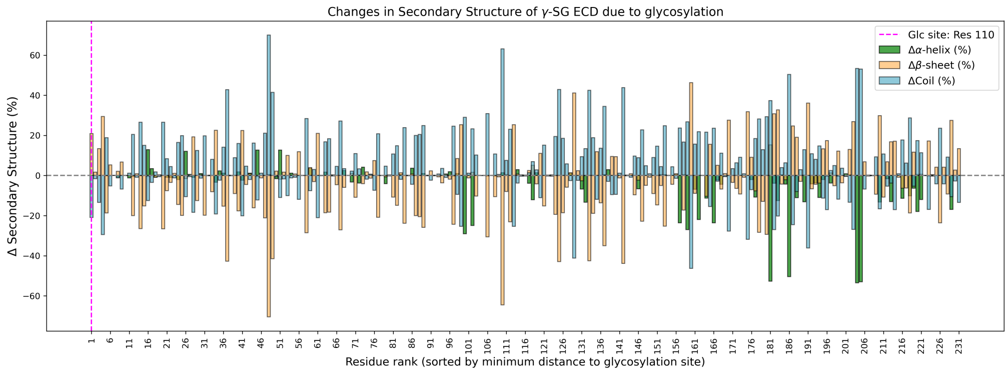

Rank — Residue Cross-Reference Table

Rank	Residue	Rank	Residue	Rank	Residue	Rank	Residue
1	110	59	171	117	196	175	243
2	129	60	184	118	225	176	240
3	109	61	139	119	190	177	256
4	111	62	115	120	150	178	269
5	103	63	80	121	151	179	210
6	107	64	79	122	153	180	270
7	128	65	144	123	123	181	281
8	104	66	146	124	156	182	259
9	108	67	148	125	69	183	218
10	127	68	172	126	163	184	211
11	93	69	62	127	161	185	253
12	102	70	98	128	167	186	284
13	130	71	149	129	216	187	242
14	101	72	195	130	121	188	258
15	112	73	170	131	223	189	271
16	89	74	175	132	224	190	219
17	86	75	188	133	192	191	207
18	105	76	142	134	162	192	221
19	87	77	71	135	122	193	252
20	113	78	73	136	67	194	257
21	106	79	197	137	200	195	241
22	94	80	72	138	58	196	205
23	114	81	70	139	165	197	239
24	88	82	120	140	166	198	249
25	85	83	187	141	279	199	206
26	90	84	75	142	68	200	247
27	84	85	178	143	280	201	230
28	95	86	169	144	65	202	229
29	132	87	74	145	157	203	272
30	83	88	137	146	226	204	282
31	126	89	182	147	158	205	283
32	145	90	116	148	159	206	209
33	141	91	177	149	66	207	277
34	97	92	186	150	164	208	268
35	82	93	176	151	245	209	273
36	133	94	117	152	202	210	220
37	77	95	180	153	201	211	208
38	131	96	189	154	291	212	238
39	173	97	152	155	160	213	237
40	81	98	181	156	227	214	261
41	96	99	61	157	288	215	260
42	100	100	59	158	212	216	236
43	134	101	193	159	286	217	248
44	147	102	60	160	217	218	235
45	92	103	215	161	222	219	234
46	185	104	194	162	289	220	232
47	78	105	124	163	203	221	231
48	183	106	199	164	255	222	267
49	76	107	63	165	285	223	275
50	99	108	198	166	287	224	276
51	91	109	118	167	228	225	262
52	125	110	191	168	254	226	265
53	140	111	214	169	204	227	274
54	136	112	155	170	250	228	263
55	174	113	64	171	244	229	233
56	138	114	168	172	251	230	266
57	143	115	179	173	246	231	264
58	119	116	154	174	278		

Figure S6: Glycosylation-induced changes in secondary structure of monomer γ-SG ECD. Difference in secondary structure content ($\Delta\%$) between glycosylated (1 Glc) and non-glycosylated systems (glycosylated - non-glycosylated) per residue, assigned using the DSSP algorithm. Residues are ranked by their minimum C_α– C_α distance to the glycosylation site (N110) over the 3μs trajectory. The cross-reference table provides the correspondence between rank and residue number.

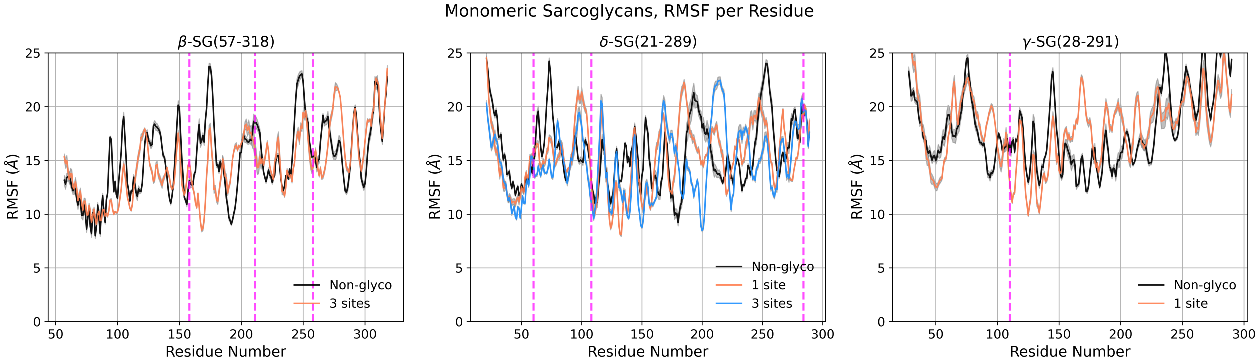

Figure S7: RMSF of C_α atoms for the β-SG (left), δ-SG (middle), and γ-SG (right) subunits. Shaded regions in grey represent $\pm$ standard deviation estimated from the trajectory chunking method (see S1.5.1)

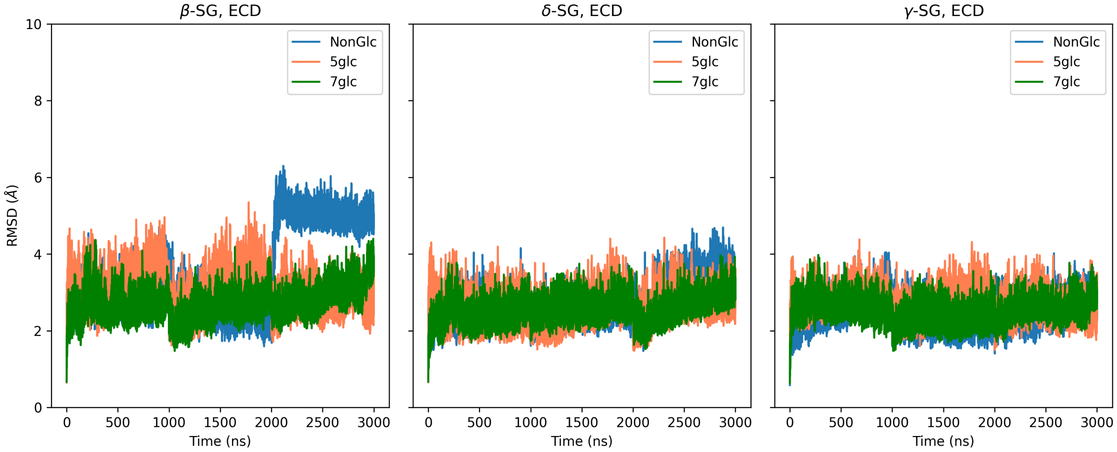

Figure S8: RMSD comparison between glycosylated (5- and 7-site: 5Glc and 7Glc, respectively) and nonglycosylated (NonGlc) forms of the SG complex over a 3μs accumulated trajectory. The right panel shows β-SG, the middle panel δ-SG, and the left panel γ-SG components of the complex.

Table S6: Glycosylation site information and RMSF differences at the glycosylation site, when all 7 reported glycosylation sites are glycosylated.
$\Delta\text{RMSF}_i = \text{RMSF}_{i,P} - \text{RMSF}_{i,GP}$, where i is for the glycosylated site, **GP** for glycosylated protein, and **P** for the nonglycosylated protein.

Name	# of glycans	Glycosylation site	# of sugar residues	Location of glycosylation site	ΔRMSF_i	Avg. | ΔRMSF |
β-SG	3	ASN-158	2	loop	0.1642	0.1299
		ASN-211	3	loop	0.2037	
		ASN-258	3	β-sheet	0.0218	
δ-SG	3	ASN-60	3	loop	-0.1554	0.6590
		ASN-108	3	loop	0.0102	
		ASN-284	3	loop	1.8115	
γ-SG	1	ASN-110	3	loop	-0.1829	-

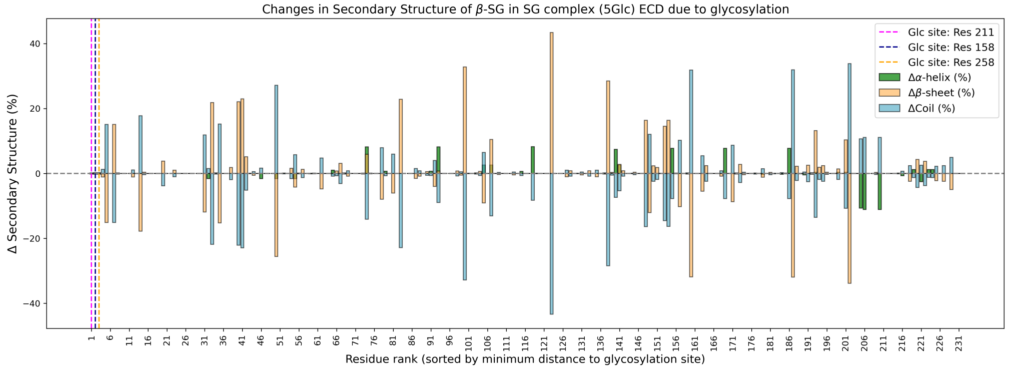

Rank — Residue Cross-Reference Table

Rank	Residue	Rank	Residue	Rank	Residue	Rank	Residue
1	211	59	249	117	165	175	178
2	158	60	236	118	266	176	172
3	258	61	233	119	134	177	302
4	210	62	237	120	149	178	126
5	259	63	137	121	226	179	278
6	159	64	240	122	267	180	187
7	257	65	315	123	308	181	132
8	212	66	316	124	148	182	120
9	157	67	253	125	228	183	173
10	214	68	219	126	169	184	279
11	156	69	251	127	201	185	131
12	213	70	152	128	190	186	186
13	256	71	241	129	170	187	283
14	209	72	205	130	221	188	277
15	260	73	193	131	298	189	272
16	215	74	264	132	225	190	121
17	216	75	206	133	166	191	112
18	261	76	296	134	181	192	280
19	160	77	146	135	229	193	275
20	155	78	288	136	180	194	276
21	141	79	314	137	227	195	176
22	143	80	232	138	309	196	281
23	293	81	250	139	129	197	177
24	161	82	203	140	183	198	282
25	142	83	287	141	305	199	273
26	255	84	136	142	245	200	111
27	140	85	231	143	244	201	274
28	208	86	247	144	304	202	125
29	217	87	199	145	285	203	110
30	154	88	252	146	311	204	174
31	139	89	248	147	223	205	124
32	292	90	147	148	269	206	122
33	262	91	313	149	128	207	175
34	318	92	204	150	117	208	109
35	294	93	265	151	222	209	99
36	144	94	164	152	133	210	123
37	254	95	191	153	189	211	100
38	196	96	151	154	310	212	108
39	207	97	168	155	184	213	98
40	295	98	167	156	130	214	107
41	263	99	192	157	115	215	101
42	218	100	242	158	118	216	90
43	197	101	220	159	127	217	97
44	138	102	200	160	299	218	91
45	145	103	297	161	301	219	103
46	291	104	150	162	179	220	102
47	162	105	307	163	270	221	89
48	239	106	202	164	171	222	106
49	153	107	306	165	300	223	105
50	289	108	135	166	113	224	104
51	194	109	230	167	119	225	96
52	238	110	182	168	303	226	93
53	195	111	243	169	185	227	92
54	235	112	286	170	114	228	88
55	290	113	246	171	116	229	95
56	163	114	268	172	271	230	94
57	234	115	312	173	188	231	87
58	198	116	224	174	284		

Figure S9: Glycosylation-induced changes in secondary structure of ECD β-SG in SG-complex. Difference in secondary structure content ($\Delta\%$) between glycosylated (5Glc in the complex) and non-glycosylated systems (glycosylated - non-glycosylated) per residue, assigned using the DSSP algorithm. Residues are ranked by their minimum C_α– C_α distance to the glycosylation site (N158, N211 and N258) over the 3μs trajectory. The cross-reference table provides the correspondence between rank and residue number.

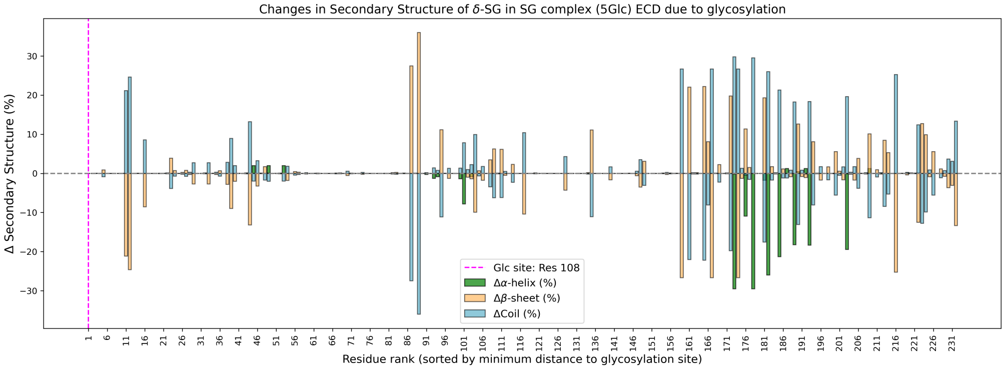

Rank — Residue Cross-Reference Table

Rank	Residue	Rank	Residue	Rank	Residue	Rank	Residue
1	108	59	80	117	175	175	223
2	109	60	152	118	172	176	284
3	107	61	150	119	63	177	231
4	127	62	96	120	176	178	286
5	126	63	153	121	177	179	232
6	105	64	149	122	178	180	233
7	110	65	78	123	179	181	283
8	128	66	79	124	180	182	287
9	125	67	98	125	181	183	289
10	106	68	141	126	182	184	234
11	104	69	156	127	185	185	282
12	129	70	154	128	183	186	235
13	131	71	97	129	184	187	266
14	130	72	148	130	186	188	236
15	111	73	77	131	195	189	281
16	103	74	68	132	194	190	267
17	124	75	155	133	193	191	237
18	132	76	142	134	192	192	265
19	123	77	76	135	197	193	280
20	112	78	157	136	190	194	273
21	133	79	158	137	191	195	271
22	102	80	147	138	188	196	274
23	134	81	143	139	196	197	240
24	120	82	69	140	202	198	269
25	122	83	75	141	200	199	256
26	113	84	159	142	203	200	272
27	121	85	145	143	187	201	278
28	118	86	67	144	199	202	268
29	85	87	70	145	201	203	279
30	101	88	146	146	198	204	257
31	87	89	74	147	204	205	258
32	135	90	160	148	189	206	238
33	84	91	166	149	205	207	241
34	83	92	144	150	206	208	242
35	114	93	168	151	207	209	264
36	119	94	167	152	208	210	244
37	88	95	71	153	209	211	239
38	86	96	59	154	210	212	243
39	93	97	60	155	211	213	270
40	100	98	72	156	212	214	263
41	82	99	66	157	213	215	245
42	138	100	169	158	218	216	246
43	89	101	58	159	229	217	254
44	90	102	163	160	216	218	253
45	117	103	170	161	220	219	275
46	136	104	164	162	214	220	277
47	81	105	165	163	219	221	255
48	94	106	65	164	217	222	276
49	115	107	73	165	221	223	259
50	92	108	161	166	227	224	262
51	137	109	61	167	228	225	261
52	95	110	57	168	225	226	260
53	116	111	64	169	226	227	251
54	91	112	162	170	224	228	252
55	139	113	62	171	215	229	247
56	99	114	171	172	222	230	248
57	151	115	173	173	285	231	250
58	140	116	174	174	230	232	249

Figure S10: Glycosylation-induced changes in secondary structure of ECD δ-SG in SG-complex. Difference in secondary structure content (Δ%) between glycosylated (5Glc in the complex) and non-glycosylated systems (glycosylated - non-glycosylated) per residue, assigned using the DSSP algorithm. Residues are ranked by their minimum C_α– C_α distance to the glycosylation site (N108) over the 3μs trajectory. The cross-reference table provides the correspondence between rank and residue number.

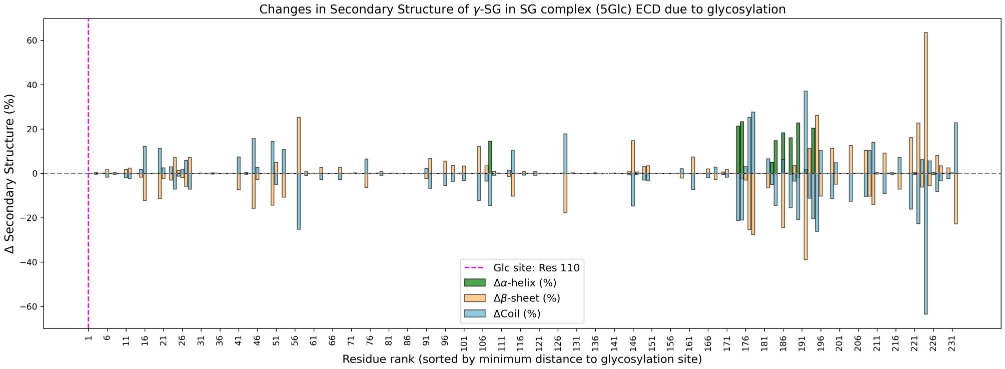

Rank — Residue Cross-Reference Table

Rank	Residue	Rank	Residue	Rank	Residue	Rank	Residue
1	110	59	153	117	175	175	287
2	111	60	142	118	176	176	270
3	109	61	154	119	187	177	238
4	129	62	155	120	177	178	239
5	107	63	81	121	188	179	227
6	128	64	99	122	186	180	228
7	130	65	152	123	189	181	266
8	108	66	100	124	179	182	271
9	112	67	143	125	190	183	284
10	127	68	80	126	178	184	283
11	106	69	151	127	185	185	241
12	131	70	156	128	180	186	281
13	133	71	158	129	181	187	229
14	132	72	157	130	184	188	289
15	105	73	144	131	191	189	237
16	113	74	145	132	192	190	286
17	126	75	79	133	183	191	236
18	134	76	149	134	193	192	285
19	125	77	150	135	182	193	230
20	114	78	159	136	195	194	282
21	135	79	70	137	194	195	265
22	104	80	147	138	197	196	272
23	136	81	71	139	196	197	234
24	121	82	78	140	198	198	242
25	122	83	160	141	199	199	231
26	124	84	168	142	201	200	233
27	123	85	148	143	200	201	280
28	115	86	161	144	202	202	235
29	103	87	69	145	203	203	246
30	137	88	146	146	204	204	243
31	120	89	169	147	213	205	245
32	89	90	167	148	205	206	273
33	117	91	77	149	216	207	244
34	90	92	72	150	212	208	232
35	119	93	166	151	214	209	264
36	86	94	165	152	211	210	279
37	87	95	162	153	215	211	247
38	88	96	76	154	219	212	274
39	85	97	68	155	210	213	275
40	116	98	73	156	217	214	263
41	102	99	170	157	209	215	248
42	118	100	61	158	218	216	278
43	95	101	67	159	221	217	276
44	140	102	74	160	220	218	277
45	91	103	172	161	206	219	262
46	92	104	164	162	207	220	249
47	84	105	62	163	208	221	261
48	83	106	66	164	222	222	250
49	97	107	171	165	223	223	260
50	138	108	60	166	269	224	251
51	96	109	163	167	268	225	258
52	139	110	173	168	224	226	259
53	93	111	75	169	226	227	252
54	94	112	64	170	291	228	253
55	101	113	63	171	240	229	256
56	98	114	174	172	225	230	257
57	82	115	59	173	267	231	255
58	141	116	65	174	288	232	254

Figure S11: Glycosylation-induced changes in secondary structure of ECD γ-SG in SG-complex. Difference in secondary structure content (Δ%) between glycosylated (5Glc in the complex) and non-glycosylated systems (glycosylated - non-glycosylated) per residue, assigned using the DSSP algorithm. Residues are ranked by their minimum C_α– C_α distance to the glycosylation site (N110) over the 3μs trajectory. The cross-reference table provides the correspondence between rank and residue number.

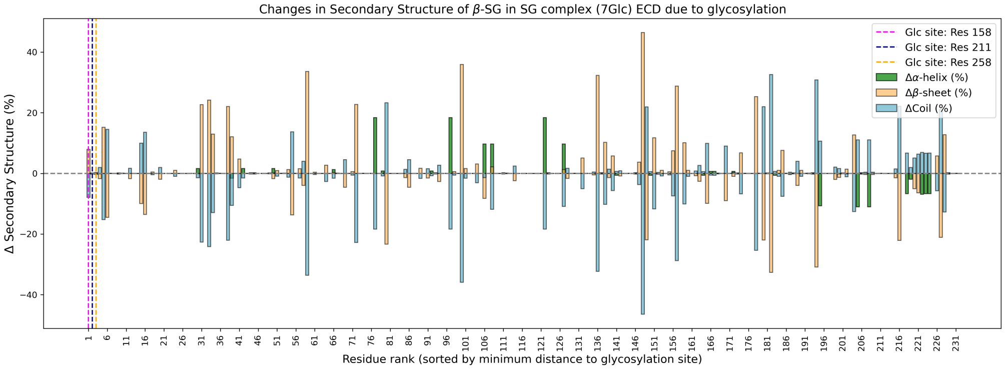

Rank — Residue Cross-Reference Table

Rank	Residue	Rank	Residue	Rank	Residue	Rank	Residue
1	158	59	288	117	226	175	178
2	211	60	194	118	165	176	172
3	258	61	219	119	268	177	187
4	210	62	240	120	148	178	271
5	257	63	233	121	149	179	173
6	259	64	253	122	266	180	283
7	159	65	195	123	228	181	126
8	157	66	315	124	267	182	120
9	212	67	236	125	225	183	186
10	214	68	152	126	169	184	132
11	156	69	251	127	305	185	272
12	213	70	198	128	201	186	282
13	256	71	206	129	134	187	121
14	216	72	287	130	221	188	131
15	215	73	137	131	170	189	112
16	209	74	241	132	166	190	176
17	261	75	296	133	190	191	177
18	260	76	205	134	181	192	273
19	160	77	264	135	180	193	281
20	155	78	146	136	308	194	125
21	141	79	314	137	285	195	124
22	143	80	250	138	229	196	174
23	161	81	247	139	227	197	111
24	293	82	232	140	309	198	110
25	142	83	248	141	183	199	276
26	255	84	193	142	245	200	278
27	217	85	203	143	223	201	274
28	208	86	252	144	244	202	280
29	154	87	231	145	311	203	175
30	292	88	136	146	222	204	277
31	262	89	147	147	300	205	122
32	140	90	164	148	269	206	279
33	294	91	199	149	299	207	275
34	139	92	313	150	184	208	123
35	318	93	297	151	304	209	99
36	254	94	204	152	117	210	109
37	144	95	168	153	129	211	100
38	295	96	220	154	118	212	98
39	289	97	265	155	128	213	108
40	207	98	167	156	310	214	107
41	218	99	151	157	270	215	101
42	291	100	242	158	133	216	91
43	162	101	150	159	189	217	97
44	263	102	191	160	171	218	103
45	196	103	286	161	179	219	90
46	145	104	192	162	127	220	106
47	153	105	200	163	284	221	102
48	239	106	307	164	185	222	89
49	197	107	182	165	115	223	105
50	290	108	306	166	301	224	104
51	316	109	312	167	302	225	93
52	138	110	202	168	130	226	95
53	238	111	230	169	119	227	96
54	235	112	246	170	116	228	92
55	249	113	224	171	113	229	88
56	163	114	298	172	303	230	94
57	234	115	135	173	114	231	87
58	237	116	243	174	188		

Figure S12: Glycosylation-induced changes in secondary structure of ECD β-SG in SG-complex. Difference in secondary structure content ($\Delta\%$) between glycosylated (7Glc in the complex) and non-glycosylated systems (glycosylated - non-glycosylated) per residue, assigned using the DSSP algorithm. Residues are ranked by their minimum C_α– C_α distance to the glycosylation site (N158, N211 and N258) over the 3μs trajectory. The cross-reference table provides the correspondence between rank and residue number.

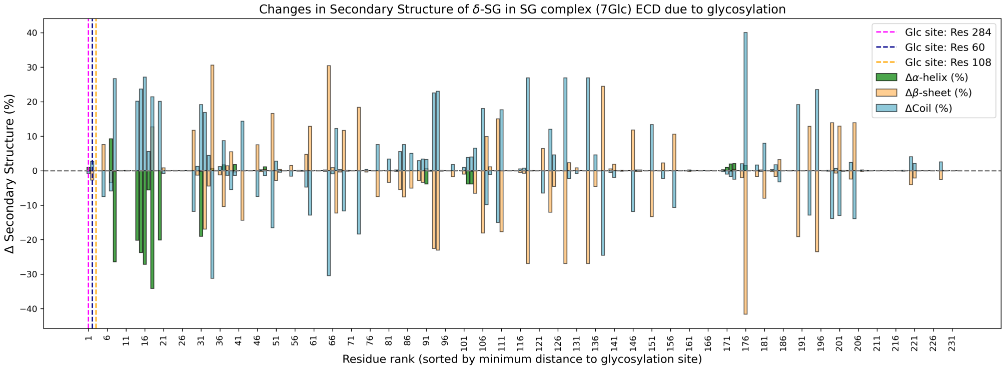

Rank — Residue Cross-Reference Table

Rank	Residue	Rank	Residue	Rank	Residue	Rank	Residue
1	284	59	260	117	234	175	223
2	60	60	71	118	230	176	170
3	108	61	123	119	99	177	163
4	109	62	112	120	253	178	207
5	61	63	78	121	139	179	205
6	59	64	270	122	151	180	165
7	283	65	258	123	140	181	164
8	285	66	267	124	235	182	208
9	107	67	269	125	236	183	204
10	127	68	133	126	252	184	171
11	57	69	259	127	152	185	161
12	67	70	81	128	229	186	209
13	66	71	102	129	238	187	174
14	281	72	82	130	150	188	162
15	287	73	268	131	239	189	218
16	286	74	77	132	96	190	175
17	58	75	134	133	153	191	216
18	282	76	256	134	228	192	173
19	105	77	76	135	240	193	222
20	280	78	120	136	237	194	217
21	126	79	72	137	149	195	210
22	62	80	83	138	250	196	172
23	110	81	85	139	251	197	203
24	68	82	122	140	154	198	215
25	128	83	257	141	248	199	220
26	125	84	113	142	98	200	219
27	289	85	119	143	141	201	211
28	106	86	87	144	156	202	212
29	273	87	121	145	97	203	176
30	65	88	101	146	227	204	202
31	279	89	86	147	241	205	221
32	104	90	84	148	242	206	177
33	263	91	117	149	245	207	213
34	274	92	135	150	155	208	214
35	69	93	114	151	249	209	178
36	265	94	118	152	148	210	201
37	276	95	88	153	247	211	200
38	278	96	255	154	226	212	179
39	272	97	75	155	157	213	199
40	275	98	93	156	142	214	198
41	63	99	73	157	246	215	180
42	129	100	232	158	158	216	181
43	131	101	100	159	143	217	182
44	130	102	116	160	147	218	192
45	271	103	115	161	243	219	185
46	64	104	90	162	225	220	197
47	266	105	138	163	145	221	183
48	277	106	89	164	244	222	191
49	111	107	136	165	159	223	193
50	70	108	94	166	146	224	184
51	103	109	254	167	144	225	190
52	264	110	74	168	160	226	194
53	124	111	231	169	224	227	186
54	132	112	92	170	166	228	189
55	80	113	95	171	167	229	196
56	79	114	233	172	169	230	195
57	262	115	137	173	168	231	188
58	261	116	91	174	206	232	187

Figure S13: Glycosylation-induced changes in secondary structure of ECD δ-SG in SG-complex. Difference in secondary structure content (Δ%) between glycosylated (7Glc in the complex) and non-glycosylated systems (glycosylated - non-glycosylated) per residue, assigned using the DSSP algorithm. Residues are ranked by their minimum C_α– C_α distance to the glycosylation site (N60, N108, N284) over the 3μs trajectory. The cross-reference table provides the correspondence between rank and residue number.

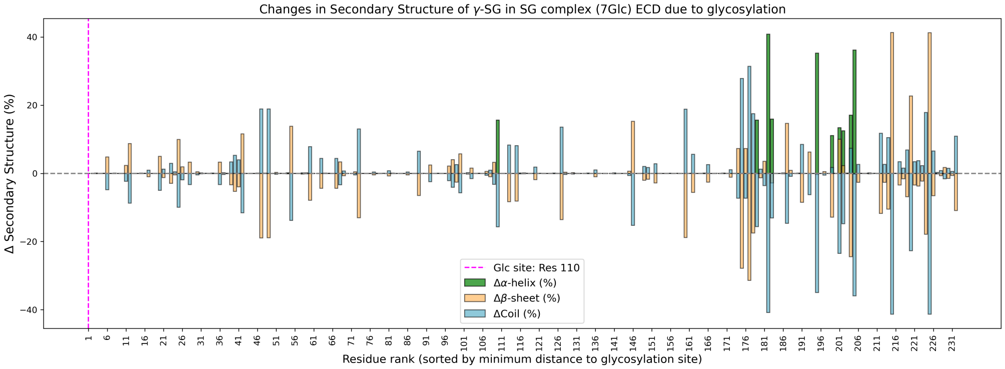

Rank — Residue Cross-Reference Table

Rank	Residue	Rank	Residue	Rank	Residue	Rank	Residue
1	110	59	118	117	175	175	239
2	111	60	153	118	176	176	231
3	109	61	155	119	187	177	238
4	129	62	154	120	177	178	240
5	107	63	81	121	188	179	288
6	128	64	100	122	189	180	270
7	130	65	152	123	179	181	237
8	108	66	99	124	186	182	284
9	112	67	80	125	190	183	287
10	127	68	156	126	178	184	267
11	106	69	143	127	180	185	235
12	131	70	151	128	185	186	236
13	132	71	158	129	181	187	291
14	133	72	157	130	184	188	243
15	105	73	79	131	191	189	242
16	134	74	145	132	183	190	241
17	113	75	144	133	192	191	233
18	126	76	150	134	182	192	245
19	125	77	149	135	193	193	232
20	114	78	159	136	195	194	246
21	135	79	71	137	194	195	283
22	104	80	147	138	197	196	244
23	136	81	70	139	196	197	271
24	124	82	78	140	198	198	234
25	123	83	160	141	199	199	285
26	122	84	161	142	201	200	266
27	103	85	168	143	200	201	286
28	115	86	148	144	202	202	289
29	137	87	169	145	203	203	247
30	89	88	146	146	204	204	281
31	90	89	77	147	214	205	282
32	86	90	167	148	205	206	272
33	87	91	69	149	213	207	280
34	85	92	72	150	216	208	265
35	88	93	162	151	215	209	248
36	121	94	166	152	212	210	273
37	116	95	165	153	211	211	278
38	95	96	68	154	210	212	279
39	92	97	76	155	209	213	264
40	102	98	73	156	217	214	275
41	91	99	66	157	219	215	249
42	93	100	62	158	206	216	277
43	83	101	170	159	218	217	274
44	140	102	61	160	221	218	263
45	84	103	67	161	220	219	276
46	97	104	74	162	207	220	250
47	138	105	164	163	208	221	262
48	120	106	172	164	222	222	259
49	96	107	163	165	223	223	260
50	94	108	173	166	224	224	261
51	119	109	171	167	226	225	251
52	139	110	60	168	225	226	258
53	101	111	75	169	227	227	257
54	117	112	64	170	228	228	256
55	82	113	63	171	269	229	255
56	141	114	65	172	229	230	252
57	98	115	174	173	268	231	253
58	142	116	59	174	230	232	254

Figure S14: Glycosylation-induced changes in secondary structure of ECD γ-SG in SG-complex. Difference in secondary structure content (Δ%) between glycosylated (7Glc in the complex) and non-glycosylated systems (glycosylated - non-glycosylated) per residue, assigned using the DSSP algorithm. Residues are ranked by their minimum C_α– C_α distance to the glycosylation site (N110) over the 3μs trajectory. The cross-reference table provides the correspondence between rank and residue number.

References

(1) McGibbon, R. T.; Beauchamp, K. A.; Harrigan, M. P.; Klein, C.; Swails, J. M.; Hernández, C. X.; Schwantes, C. R.; Wang, L.-P.; Lane, T. J.; Pande, V. S. MDTraj: A Modern Open Library for the Analysis of Molecular Dynamics Trajectories. *Biophysical Journal* **2015**, *109*, 1528 – 1532.

(2) Roe, D. R.; Cheatham III, T. E. PTRAJ and CPPTRAJ: software for processing and analysis of molecular dynamics trajectory data. *Journal of Chemical Theory and Computation* **2013**, *9*, 3084–3095.

(3) Pedregosa, F. et al. Scikit-learn: Machine Learning in Python. *Journal of Machine Learning Research* **2011**, *12*, 2825–2830.

(4) Gowers, R. J.; Linke, M.; Barnoud, J.; Reddy, T. J. E.; Melo, M. N.; Seyler, S. L.; Domanski, J.; Dotson, D. L.; Buchoux, S.; Kenney, I. M., et al. *MDAnalysis: a Python package for the rapid analysis of molecular dynamics simulations*; 2019.

(5) Michaud-Agrawal, N.; Denning, E. J.; Woolf, T. B.; Beckstein, O. MDAnalysis: a toolkit for the analysis of molecular dynamics simulations. *Journal of Computational Chemistry* **2011**, *32*, 2319–2327.

(6) Paszke, A.; Gross, S.; Massa, F.; Lerer, A.; Bradbury, J.; Chanan, G.; Killeen, T.; Lin, Z.; Gimelshein, N.; Antiga, L., et al. Pytorch: An imperative style, high-performance deep learning library. *Advances in Neural Information Processing Systems* **2019**, *32*.

(7) Hunter, J. D. Matplotlib: A 2D graphics environment. *Computing in Science & Engineering* **2007**, *9*, 90–95.

(8) Lyman, E.; Zuckerman, D. M. On the structural convergence of biomolecular simulations by determination of the effective sample size. *The Journal of Physical Chemistry B* **2007**, *111*, 12876–12882.

(9) Klem, H.; Hocky, G. M.; McCullagh, M. Size-and-shape space gaussian mixture models for structural clustering of molecular dynamics trajectories. *Journal of Chemical Theory and Computation* **2022**, *18*, 3218–3230.

(10) Sasmal, S.; Pal, T.; Hocky, G. M.; McCullagh, M. Quantifying Unbiased Conformational Ensembles from Biased Simulations Using ShapeGMM. *Journal of Chemical Theory and Computation* **2024**, *20*, 3492–3502.

(11) Sasmal, S.; McCullagh, M.; Hocky, G. M. Tutorial on quantifying and sampling biomolecular ensembles with ShapeGMM. *The Journal of Chemical Physics* **2025**, *163*, 241501.

(12) Harris, M. S.; Dolan, R. F.; Bryce, J. R.; Ewusi, J. G.; Cook, G. A. In Vitro Glycosylation of the Membrane Protein γ-Sarcoglycan in Nanodiscs. *ACS Omega* **2023**, *8*, 40904–40910.
